# A panel regression analysis for the COVID-19 epidemic in the United States

**DOI:** 10.1371/journal.pone.0273344

**Published:** 2022-08-19

**Authors:** Yinpei Guo, Bo Li, Tonghua Duan, Nan Yao, Han Wang, Yixue Yang, Shoumeng Yan, Mengzi Sun, Ling Wang, Yan Yao, Yuchen Sun, Jiwei Jia, Siyu Liu

**Affiliations:** 1 Department of Epidemiology and Biostatistics, School of Public Health, Jilin University, Changchun, China; 2 Department of Computational Mathematics, School of Mathematics, Jilin University, Changchun, China; 3 Jilin National Applied Mathematical Center, Jilin University, Changchun, China; The University of Southern Mississippi, UNITED STATES

## Abstract

This study explored the roles of epidemic-spread-related behaviors, vaccination status and weather factors during the COVID-19 epidemic in 50 U.S. states since March 2020. Data from March 1, 2020 to February 5, 2022 were incorporated into panel model. The states were clustered by the k-means method. In addition to discussing the whole time period, we also took multiple events nodes into account and analyzed the data in different time periods respectively by panel linear regression method. In addition, influence of cluster grouping and different incubation periods were been discussed. Non-segmented analysis showed the rate of people staying at home and the vaccination dose per capita were significantly negatively correlated with the daily incidence rate, while the number of long-distance trips was positively correlated. Weather indicators also had a negative effect to a certain extent. Most segmental results support the above view. The vaccination dose per capita was unsurprisingly proved to be the most significant factor especially for epidemic dominated by Omicron strains. 7-day was a more robust incubation period with the best model fit while weather had different effects on the epidemic spread in different time period. The implementation of prevention behaviors and the promotion of vaccination may have a successful control effect on COVID-19, including variants’ epidemic such as Omicron. The spread of COVID-19 also might be associated with weather, albeit to a lesser extent.

## Introduction

The rapid spread of COVID-19 had seriously affected people’s health and daily life which imposed a great burden on almost every country [1]. The COVID-19 epidemic started in December 2019 and quickly swept the world. At the beginning of 2020, the cases in the U.S. only showed a sporadic state [2, 3]. However in the early days of the epidemic, heated discussions on ‘wearing masks’ and ‘freedom and human rights’ in the American society as well as residents’ limited implementation of prevention measures resulted in uncontrollably spreading epidemic [4]. Until December 14, 2020, when the vaccine officially began to be universally popularized, a total of 16,891,386 cases had been diagnosed in the U.S., and as of February 5, 2022, this number reached 77,502,221 [5].

The COVID-19 is caused by the spread of the new coronavirus SARS-CoV-2, which can exist in the air or droplets and spread through breathing and talking when people contacting with others face to face [6–8]. In addition, this type of virus also has the characteristics of aerosol transmission [9, 10]. Compared with SARS, the fatality rate of SARS-CoV-2 was a bit lower, but its community communication power showed a higher level [11, 12]. SARS-CoV-2 mutated continuously since December 2020, and multiple SARS-CoV-2 variants have been described. Nowadays, type Delta and Omicron are widely concerned (VOCs), and their community transmission is even gradually increasing with shortening incubation period [13].

The spread of SARS-CoV-2 depends on droplets or aerosols, thus the influence of environmental factors such as temperature, humidity, rainfall, air pressure should be considered [14, 15]. Early studies showed that the survival and spread of coronavirus were affected by weather factors to a certain degree. The daily incidence rate reached its peak at around 16–28°C [16, 17]. Some experimental researches showed that high temperature and high humidity will accelerate the inactivation of the virus [18, 19]. As one of the factors which was most often been discussed, different people obtained different conclusions after conducting research on the effect of weather towards COVID-19—there existed some controversy about the effect sizes and regional characteristic of weather on epidemic developing [20, 21]. Research by Hamdan. M [22] showed that air pressure supported the virus’s spread in Amman and Zarqa. Recent study indicated that the optimal temperature for spread of COVID-19 ranging from 41–57.2°F [23]. Therefore, it is necessary to consider the role of weather in the spread of the COVID-19 epidemic appropriately.

Since COVID-19 is a highly contagious viral disease, public health prevention and mitigation policies such as social distancing, isolation, and quarantine were suggested to reduce the spread of the virus [24]. Previous study demonstrated that the number of infections averted through the use of quarantine was expected to be very low provided that isolation was effective [25, 26]. Thus, it is reasonable to say that reducing going out may be the most effective measure against infecting. But with the continuous advancement of herd immunity in the U.S., the popularization of the vaccine, as well as the mutation of the SARS-CoV-2 strains… this daily prevention measure may show a different state in slowing down the development than in the early stage of the epidemic. Therefore, it is very interesting to explore the effect of daily epidemic-spread-related behaviors in different periods. At the same time, with the promotion of vaccination work, it is also of positive significance to check the inhibitory effect of vaccines on epidemic dominated by different strains.

As a data model suitable for simultaneous observation of time and space dimensions, the panel model is very beneficial for dynamic model monitoring. Previous studies have tried to incorporate panel model into research and achieved very good results [27, 28]. For example, Guliyev [28] verified the optimal robustness of panel model results when exploring the relationship between confirmed COVID-19 cases, deaths, and recovered cases after treatment. Based on panel model, we may obtain the differences in the effect of factors on the epidemic in different spaces by integrating and analyzing the data from similar states which exist spatial dependence.

Thus, this study aims to discuss the development of the COVID-19 epidemic in 50 states in U.S. based on panel model, and analyze the relationship between the daily incidence rate (DIR) and people’s implementation of preventive measures or quarantine policy and vaccination status in the later period of the epidemic after controlling for the influence of the weather.

## Materials and methods

### Data sources

In this study, data of 50 states in U.S. were used as the research subjects. According to the data released by Johns Hopkins University [5], the DIR data of each state from March 1, 2020 to February 5, 2022 were included as the dependent variable. Independent variable data included the proportion of daily residents at home (AHR) and the daily trips were both obtained from the website of the Bureau of Transportation Statistics, and trips are defined as movements that include a stay of longer than 10 minutes at an anonymized location away from home [29]. Daily trips per capita (TR) equals to the number of travel times divided by population. We also obtained the number of medium-distance trip (TR>25 miles) and long-distance/interstate trip (TR>250 miles) per capita. Independent variable data vaccination status expressed by the daily administered vaccination dose per capita (AVD) obtained from CDC [30]. Missing values were completed by linear interpolation. Weather factors including daily temperature (T), humidity (H), wind speed (WS), air pressure (AP) and precipitation (PPTN) of every state were collected from Weather Underground website [31].

### Preprocessing

The onset of COVID-19 has a certain incubation period. Previous studies found that the effect time of exposure to coronavirus is about 5–7 days, even longer [32–34]. The result of dynamic Public Health Surveillance of U.S. COVID-19 conducted by Dr. Post [35] suggested the coefficients on the 7-day lag were both positive and statistically significant. Thus, we chose 7 days as the incubation period to preprocess the data, which means the respective variables would be correspond to the DIR after 7 days. In addition, we also took into account the fact that the SARS-CoV-2 variants may have shorter incubation period, so we also used 3-day or 10-day as the incubation period to conduct uncertainty analysis.

**Fig 1:**
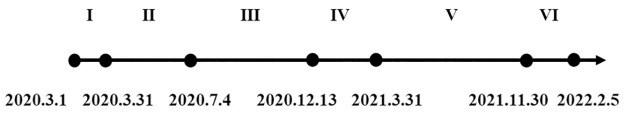
Segmentation method of time period.

In addition to analyzing the data from March 1, 2020 to February 5, 2022, we also divided the whole process into 6 different segments according to the time of quarantine policy introduction, the time of the first vaccination, the time when the mutant strain became popular in the U.S., etc. The influencing factors at each segment were explored. The segmentation method is as follows: At the end of March 2020, almost every state basically required the implementation of statewide stay-at-home orders for its residents [36, 37]. On July 4, 2020, almost the entire country opened with virtually no restrictions [38]. In late 2020, U.S. residents began to be vaccinated, and this number was recorded by the CDC from December 12, 2020 [30]. The Delta variant was first detected in March 2021 in the U.S. [13]. The first U.S. case of COVID-19 caused by the Omicron variant was first reported on December 1, 2021 [39]. The specific segmentation method was shown in Fig 1.

### Panel data model

Panel data is a set of two-dimensional cross-sectional data that contains both time and space. It can be understood as a set of data formed by intercepting certain characteristic values of *i* objects at *t* different time nodes [40]. Therefore, the panel data can be represented by double subscript variable *y*_*it*_.

$$y_{it}=\alpha_{i}+\sum_{k=1}^{k}x_{itk}\beta_{k}+u_{it}$$ (1)

*α_i_: intercept*

*i = 1,2,3…N (Number of subjects)*

*t = 1,2,3…T (Point of observation of each individual)*

*k = Number of explanatory variables*

*u_it_: random error*

We can also regard multi-indicator panel data as a composite matrix. For the same moment, the observation values of different indicators of all samples can form a time matrix. For the same indicator, several samples can be selected to observe it at each moment and these data can form an indicator matrix.

This study used a panel data model to fit the DIR of 50 states in the U.S., and considered the development of COVID-19 both in the vertical—time dimension, and the horizontal—states dimension. Through the cluster and multiple linear regression model analysis of the panel data, the characteristic of both space and time dimensions of the epidemic can be separately explored.

### Statistical analysis

Firstly, the cluster analysis of the panel data model was conducted based on the traditional classic K-Means algorithm. The data in this study can be expressed as an *n*×*d* matrix *X*, while *n* is the number of samples (*n* = 35350 in our study), *d* is the dimension of the samples (*d* = 9 in our study). *k* cluster centers are expressed as *k*×*d* matrix *C*, while *k* = 3, and each row of *C* represents a cluster center. The distance from the sample to the *k* centers is expressed as an *n*×*k* matrix *D*.

According to the optimization problem (1) to assign each sample point to the new nearest class center (2) to form *k* classes and update the sample mean of this class as the class center. Then, update the class center iteratively until the class center keep stable.


$$\underset{c_{1}\cdots c_{k}}{\text{min}}{\sum_{j=1}^{k}\sum_{i=1}^{n}\left\|x_{i}-c_{j}\right\|}_{2}^{2}$$ (2)

$$c_{j}^{\left(i\right)}=\frac{1}{n}\overset{n}{\underset{i=1}{\sum }}x_{i}$$ (3)

Group visualization is completed according to the maximum number of days that each research object belongs to a certain category in the research time. For example, according to our study, Alaska (AK) had most days in cluster 3, thus, we classified it into the third category. Analysis of every one of three category was completed in order to test the impact of clustering results and explore the effect of factors among similar states.

The Hausman test was used to select random effects model or fixed effects model for panel regression analysis. And the fitting of the linear regression of the panel data model performed by the ordinary least square method (OLS). While based on the characteristics of panel data: the disturbance items between different individuals are independent of each other, but there is often autocorrelation among the disturbance items of the same individual in different periods, so we used the robust command to perform regression analysis under the clustering robust standard error to reduce the overestimation of the influence of the independent variable on the dependent variable to obtain a more accurate linear trend.

The python-based software code and Stata16.0 were used for analysis. α = 0.05.

## Results

### Cluster and basic situation

**Fig 2:**
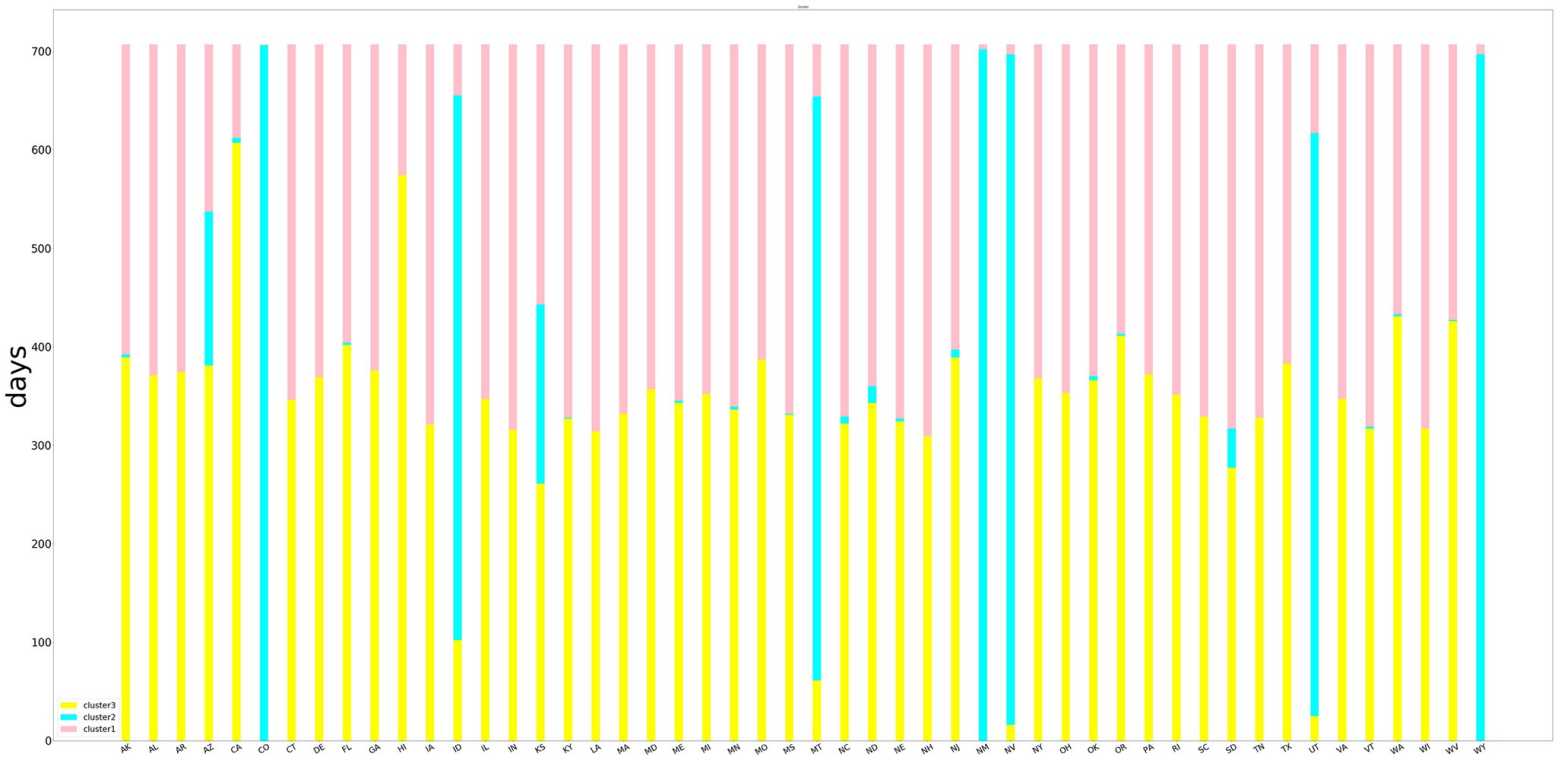
The frequency distribution cluster graph.

The above independent variables were used to cluster the 50 states, and the frequency distribution cluster graph was shown in Fig 2. The first category contained the states with characteristics of lower daily AHR, TR (>250 miles), AVD, T, moderate TR, H, PPTN, higher TR (>25 miles), AP, and WS represented by Iowa (IA) and New Hampshire (NH), contains 23 states. The second category were the states contains 7 states represented by Colorado (CO) and New Mexico (NM), which with low daily H, AP, PPTN, high AHR, TR, TR (>250 miles), moderate TR (>25 miles), AVD, T and WS. The third category was represented by West Virginia (WV) and Hawaii (HI), containing the rest 20 states with low daily TR, TR (>250 miles), TR (>25 miles), WS, high AHR, AVD, T, H, AP and PPTN. The basic information of factors of these 6 states was shown in Table 1.

**Table 1:** The basic information of 6 representative states [X ± SD/M (P_25_, P_75_)].

Variable	Iowa	New Hampshire	Colorado	New Mexico	West Virginia	Hawaii
**AHR**	0.21(0.20,0.23)	0.18(0.17,0.20)	0.27(0.25,0.30)	0.25(0.23,0.27)	0.29(0.28,0.31)	0.24(0.23,0.27)
**TR**	3.76±0.69	3.87±0.71	4.07±0.80	3.92±0.70	2.76±0.55	3.00±0.62
**>25 miles**	0.36±0.05	0.35±0.05	0.28±0.04	0.33±0.04	0.23±0.03	0.08±0.02
**>250 miles**	0.007±0.003	0.004±0.001	0.01±0.004	0.01±0.003	0.008±0.002	0.005±0.001
**AVD**	0.14(0.28, 0.57)	0.12(0.29, 0.63)	0.19(0.35, 0.63)	0.18(0.35, 0.63)	0.21(0.35, 0.63)	0.22(0.42, 0.64)
**T (℉)**	50.30±21.71	52.72±18.03	53.02±18.17	57.85±15.81	56.43±15.90	75.46±4.188
**H (%)**	73.6(63.4, 83.5)	63.3(51.8, 74.9)	42.9(32.7, 56.7)	36.3(26.9, 47.5)	75.6(66.6, 82.6)	73.0(64.9, 79.7)
**WS (mph)**	10.06±4.16	6.79±3.32	9.81±3.31	8.57±3.83	4.32±2.62	7.63±2.96
**AP (Hg)**	29.06±0.20	29.73±0.23	24.14±0.35	24.47±0.43	29.00±0.23	30.01±0.07
**PPTN (in, daily)**	0.075	0.107	0.033	0.014	0.132	0.263

AHR, the proportion of daily residents at home; TR, Daily trips per capita; AVD, daily administered vaccination dose per capita; T, temperature; H, humidity; WS, wind speed; AP, air pressure; PPTN, precipitation.

### Multivariate analysis

After completing the Hausman test, the fixed-effects model was selected for multivariate regression analysis. TR (>250 miles) was more stable in all models than TR or TR (>25 miles), so Table 2 showed the results of including TR (>250 miles) as an independent variable in the model. Model results involving TR or TR (>25 miles) were presented in the S2 and S3 Tables.

**Table 2:** Multivariate analysis of influencing factors—DIR postponed for 7 days.

Variables	50 states			the first category			the second category			the third category		
	Coef.	*P*	R^2^	Coef.	*P*	R^2^	Coef.	*P*	R^2^	Coef.	*P*	R^2^
**Unsegmented**												
AHR	**-1.89E-01**	0.000	0.44	**-1.32E-01**	0.000	0.36	**-2.21E-01**	0.000	0.55	**-1.96E-01**	0.000	0.45
TR(>250 miles)	**8.42E-01**	0.000		**1.24E+00**	0.000		**4.72E-01**	0.001		**7.80E-01**	0.000	
AVD	**-2.29E+00**	0.000		**-2.59E+00**	0.000		**-3.05E+00**	0.000		**-2.18E+00**	0.000	
T	**-5.02E-04**	0.000		**-5.57E-04**	0.000		**-5.67E-04**	0.000		**-4.99E-04**	0.000	
H	9.80E-06	0.194		3.66E-05	0.141		**-6.12E-05**	0.002		**4.14E-05**	0.000	
WS	**-1.09E-04**	0.005		**-6.88E-04**	0.000		**-3.10E-04**	0.000		4.17E-05	0.363	
AP	**-2.47E-03**	0.000		**-2.39E-02**	0.000		**-1.78E-03**	0.000		**-2.09E-03**	0.000	
PPTN	**-2.12E-03**	0.000		**-3.15E-03**	0.033		**-2.29E-02**	0.000		**-2.52E-03**	0.000	
constant	1.64E-01	0.000		7.89E-01	0.000		1.59E-01	0.000		1.53E-01	0.000	
**Segmented**												
**I**												
AHR	**-5.25E-02**	0.000	0.83	**-5.77E-02**	0.000	0.89	**-7.51E-02**	0.000	0.86	**-4.73E-02**	0.000	0.83
TR(>250 miles)	**-8.97E-03**	0.000		-3.46E-03	0.182		-2.73E-02	0.377		**-8.24E-03**	0.000	
T	**2.99E-05**	0.003		4.75E-05	0.107		-4.90E-05	0.188		**7.30E-05**	0.000	
H	**-9.64E-06**	0.045		**-5.07E-05**	0.000		**-4.67E-05**	0.002		**1.46E-05**	0.012	
WS	2.91E-05	0.244		**-2.25E-04**	0.003		**2.85E-04**	0.002		**6.21E-05**	0.019	
AP	**-1.53E-03**	0.000		**-9.30E-03**	0.000		1.08E-03	0.592		**-9.50E-04**	0.002	
PPTN	**-2.09E-03**	0.000		2.14E-03	0.058		-4.31E-03	0.198		**-2.24E-03**	0.000	
constant	8.99E-02	0.000		3.29E-01	0.000		3.60E-02	0.473		6.55E-02	0.000	
**II**												
AHR	**-2.72E-02**	0.000	0.18	**-3.73E-02**	0.000	0.30	**-3.26E-02**	0.000	0.20	**-2.35E-02**	0.000	0.18
TR(>250 miles)	**1.91E-01**	0.000		**2.04E-01**	0.003		**2.62E-01**	0.000		**1.66E-01**	0.000	
T	**6.72E-05**	0.000		**1.34E-04**	0.000		**6.73E-05**	0.000		**5.78E-05**	0.000	
H	**1.33E-05**	0.000		**5.15E-05**	0.000		-1.11E-05	0.435		**1.66E-05**	0.000	
WS	**-7.78E-05**	0.000		-2.50E-05	0.710		-6.64E-05	0.252		**-9.19E-05**	0.000	
AP	**-2.24E-04**	0.003		**4.43E-03**	0.003		-2.02E-04	0.062		**-7.65E-04**	0.008	
PPTN	3.30E-04	0.136		5.44E-04	0.263		9.53E-04	0.644		5.33E-05	0.838	
constant	3.01E-02	0.000		-1.06E-01	0.017		3.29E-02	0.000		4.39E-02	0.000	
**III**												
AHR	**-1.80E-01**	0.000	0.78	**-1.88E-01**	0.000	0.80	**-1.53E-01**	0.000	0.83	**-1.85E-01**	0.000	0.77
TR(>250 miles)	**6.80E-03**	0.000		8.42E-05	0.987		**1.39E-02**	0.002		**7.24E-03**	0.000	
T	**-1.95E-04**	0.000		**-2.04E-04**	0.000		**-1.81E-04**	0.000		**-1.90E-04**	0.000	
H	**-1.62E-05**	0.000		**3.93E-05**	0.010		**3.69E-05**	0.001		**-1.39E-05**	0.015	
WS	2.01E-05	0.301		**-1.57E-04**	0.016		5.10E-05	0.267		**4.66E-05**	0.044	
AP	**1.00E-03**	0.000		**3.48E-03**	0.000		**1.36E-03**	0.000		**8.90E-04**	0.000	
PPTN	**7.03E-04**	0.006		-1.01E-03	0.052		**-2.91E-02**	0.000		**1.37E-03**	0.000	
constant	1.33E-01	0.000		7.29E-02	0.019		9.91E-02	0.000		1.38E-01	0.000	
**IV**												
AHR	**-5.87E-02**	0.000	0.69	**-2.73E-02**	0.026	0.73	-1.31E-02	0.164	0.72	**-6.87E-02**	0.000	0.70
TR(>250 miles)	-5.01E-02	0.131		1.14E-01	0.255		-3.02E-02	0.689		-6.66E-02	0.087	
AVD	**-1.05E+00**	0.000		**-2.35E+00**	0.000		**-1.51E+00**	0.000		**-8.99E-01**	0.000	
T	**1.38E-04**	0.000		**6.66E-05**	0.002		**5.23E-05**	0.006		**1.42E-04**	0.000	
H	**1.16E-05**	0.033		**-3.55E-05**	0.045		1.46E-05	0.349		**1.96E-05**	0.002	
WS	**-1.89E-04**	0.000		**-5.40E-04**	0.000		-1.08E-05	0.847		**-2.05E-04**	0.000	
AP	**-9.62E-04**	0.006		**-7.19E-03**	0.000		**5.97E-03**	0.000		**-1.55E-03**	0.000	
PPTN	**-1.81E-03**	0.000		**-5.59E-03**	0.000		-3.62E-03	0.376		**-1.61E-03**	0.000	
constant	9.29E-02	0.000		2.52E-01	0.000		-1.15E-01	0.000		1.17E-01	0.000	
**V**												
AHR	**-4.51E-02**	0.000	0.18	**-6.87E-02**	0.000	0.51	**-1.78E-01**	0.000	0.28	**-4.48E-02**	0.000	0.19
TR(>250 miles)	**2.26E-01**	0.000		**7.65E-01**	0.000		**2.91E-01**	0.028		1.86E-02	0.770	
AVD	**-5.44E-01**	0.000		**-1.73E-05**	0.000		**-8.79E-01**	0.000		**-6.70E-01**	0.000	
T	**-2.32E-04**	0.000		**-1.16E-04**	0.000		**-2.40E-04**	0.000		**-2.67E-04**	0.000	
H	**7.59E-05**	0.000		**2.09E-04**	0.000		**-3.78E-05**	0.036		**1.06E-04**	0.000	
WS	**-7.08E-04**	0.000		**-1.35E-03**	0.000		**-7.90E-04**	0.000		**-5.77E-04**	0.000	
AP	**-3.29E-03**	0.000		**-9.53E-03**	0.000		-5.95E-04	0.216		**-5.08E-03**	0.000	
PPTN	**-2.54E-03**	0.000		**-4.33E-03**	0.000		**-9.49E-03**	0.001		**-3.04E-03**	0.000	
constant	1.37E-01	0.000		3.56E-01	0.000		9.51E-02	0.000		1.92E-01	0.000	
**VI**												
AHR	**-1.66E-01**	0.000	0.21	9.56E-02	0.581	0.11	**-5.75E-01**	0.000	0.34	-6.73E-02	0.133	0.27
TR(>250 miles)	**2.26E+00**	0.000		**3.63E+00**	0.019		1.50E-01	0.888		**3.48E+00**	0.000	
AVD	**-2.32E+00**	0.000		-1.90E+00	0.184		**-6.44E+00**	0.000		**-2.13E+00**	0.000	
T	**-6.63E-04**	0.000		-4.36E-04	0.002		**4.82E-04**	0.000		**-7.27E-04**	0.000	
H	**3.09E-04**	0.000		1.91E-05	0.871		**5.40E-04**	0.000		**3.82E-04**	0.000	
WS	2.57E-04	0.071		7.56E-04	0.286		**-1.91E-03**	0.000		**9.36E-04**	0.000	
AP	**1.62E-03**	0.007		**-7.11E-03**	0.266		**-8.83E-03**	0.000		**5.68E-03**	0.000	
PPTN	**-9.44E-03**	0.000		-6.48E-03	0.400		-3.96E-02	0.050		**-9.11E-03**	0.000	
constant	2.17E-02	0.261		2.22E-01	0.305		3.75E-01	0.000		-1.39E-01	0.000	

AHR, the proportion of daily residents at home; TR, Daily trips per capita; AVD, daily administered vaccination dose per capita; T, temperature; H, humidity; WS, wind speed; AP, air pressure; PPTN, precipitation.

According to the unsegmented results, AHR, AVD and DIR were significant negatively correlated, the coefficient of T, WS, AP, PPTN was rather small, but also negatively correlated with DIR. TR (>250 miles) had a significant negative effect on DIR (Table 2). The linear regression equation was written as:

$$\begin{array}{l} DIR_{us}=-1.89E-01\times AHR+8.42E-01\times TR\left(>250\;miles\right)-2.29E+00\times AVD\\ -5.02E-04\times T-1.09E-04\times WS-2.47E-03\times AP-2.12E-03\times PPTN+1.64e-01 \end{array}$$

The regression results of the three categories after clustering were also listed in Table 2, basically consisted with the unsegmented results. TR (>250 miles) had a stronger effect on the DIR of the first category with a higher regression coefficient, while the second category was less affected by it, but vaccine had a strong inhibition on the increase of DIR (coefficient = -3.05E+00). The results of the third category were closest to the results of the 50 states, whose models also had similar R-squares.

In the first segment, in addition to the relatively significant effect of AHR on DIR, other independent variable such as TR (>250 miles) and weather indicators had a little bit effect on DIR. TR (>250 miles) even appeared weird negative correlation with DIR, while in the first and second category, it was not significant. However, from the second segment, the relationship between TR (>250 miles) and DIR became much more normal. The effect of weather on DIR was weak, T and DIR showed a positive correlation which was different from the unsegmented results.


$$\begin{array}{l} DIR_{s1}=-5.25E-02\times AHR-8.97E-03\times TR\left(>250\;miles\right)\\ +2.99E-05\times T-9.64E-06\times H-1.53E-03\times AP-2.09E-03\times PPTN+8.99E-02 \end{array}$$

In the second segment, the positive effect of TR (>250 miles) on DIR was even much higher than that of AHR (1.91E-01>2.72E-02). Similar to the first segment, the effect of weather on DIR was also weak, but a statistical association could be found, with both T and H positively contributing to DIR in this segment. The results of the three classification models were basically the same.


$$\begin{array}{l} DIR_{s2}=-2.72E-02\times AHR+1.91E-01\times TR\left(>250\;miles\right)\\ +6.72E-05\times T+1.33E-05\times H-7.78E-05\times WS-2.24E-04\times AP+3.01E-02 \end{array}$$

The third segment was the time after the full unblocking and before vaccination, and the effect of AHR on DIR was significantly higher than that of TR (>250 miles). The first category of results didn’t show a significant association between TR (>250 miles) with DIR.


$$\begin{array}{l} DIR_{s3}=-1.80E-01\times AHR+6.80E-03\times TR\left(>250\;miles\right)\\ +1.95E-04\times T-1.62E-05\times H+1.00E-03\times AP+7.03E-04\times PPTN+1.33E-01 \end{array}$$

In the fourth segment, AHR was still negatively correlated with DIR, while the effect of the vaccination was the most significant—its coefficient reached -1.05E+00, this negative effect was even more obvious in the first and second category models. The effect of TR (>250 miles) on DIR was not found.


$$\begin{array}{l} DIR_{s4}=-5.87E-02\times AHR-1.05E+00\times AVD\\ +1.38E-04\times T+1.16E-05\times H-1.89E-04\times WS-9.62E-04\times AP-1.81E-03\times PPTN+9.29E-02 \end{array}$$

In the fifth segment, the negative effect of vaccination on DIR was slightly higher than the positive effect of TR (>250 miles) on DIR (5.44E-01>2.26E-01), and both of them were higher than the inhibitory effect of AHR on DIR. The results of the second category model were the closest to the overall model, the effect of vaccination in the first category model was relatively slight, DIR was mainly affected by TR (>250 miles).


$$\begin{array}{l} DIR_{s5}=-4.51E-02\times AHR+2.26E-01\times TR\left(>250\;miles\right)-5.44E-01\times AVD\\ -2.32E-04\times T+7.59E-05\times H-7.08E-04\times WS-3.29E-03\times AP-2.54E-03\times PPTN+1.37E-01 \end{array}$$

In the last segment, both vaccination and TR (>250 miles) had significantly higher effects on DIR than AHR. As in the previous two periods, the regression coefficient for vaccination was higher than that of TR (>250 miles) (2.32E+00>2.26E+00). In the first category model, DIR was also dominated by TR (>250 miles), while the effect of vaccination on DIR was not significant. However, the results of the second category model showed that DIR in these states was significantly affected by vaccination (reached up to 6.44E+00 high) but not TR.


$$\begin{array}{l} DIR_{s6}=-1.66E-01\times AHR+2.26E+00\times TR\left(>250\;miles\right)-2.32E+00\times AVD\\ -6.63E-04\times T+3.09E-04\times H+1.62E-03\times AP-9.44E-03\times PPTN+2.17E-02 \end{array}$$

The results of the model under 3-day or 10-day incubation period were shown in the S1–S3 Tables. According to it, the R-square performance of the models under these two incubation periods was generally lower than that of the model under the 7-day incubation period.

Moreover, the fitting results of the 3-day incubation period model for AHR were not stable enough, and the 10-day incubation period model may underestimate the effect of vaccination compared with 7-day. Besides, it was worth noting that in the latter three segments, the effect of vaccination under the 7-day incubation period on DIR was consistently higher than that from model under 3-day incubation period.

## Discussion

Our study explored the roles of epidemic-spread-related behaviors and vaccination status in different segments of COVID-19 development, and used panel model clustering and liner regression to explore how these roles differ across spatial dimensions. Besides, compared different incubation periods’ model fit to observe the optimal incubation period.

With the normalization of the epidemic, the ways to prevent transmission have become well known. The significant negative correlation between AHR, AVD and DIR and the significant positive correlation between TR and DIR found by the unsegmented regression model all verified without exception that the most effective ways of epidemic prevention were staying at home, reducing the number of trips (especially long-distance interstate travel) and vaccinations, etc. (Table 2). The first segment was March 2020—a period when the epidemic had not yet fully caught on. During this period, there might not be enough cases to observe the real effect of travel times due to insufficient awareness of COVID-19 and limited testing. But under this premise, a slight association between AHR and DIR was found. The impact of travel became significant in the second segment—when all 50 states became acutely aware of the dangers of COVID-19 and enacted stay-at-home orders. We all know that after a month of quarantine, U.S. was gradually unblocking even though the outbreak was not effectively contained [38]. Thus, in the third segment, we were able to see the significant effect of AHR and travel on the epidemic, and compared with the second segment, AHR played a more important role. This might be due to the increasing awareness of COVID-19 which had indeed reduced the frequency of interstate travel. Therefore, more effective prevention behaviors—staying at home had become the main influencing factor of DIR at this stage.

In the middle and late stages of the epidemic, vaccines came out, and the American people begun to be vaccinated voluntarily or compulsorily since December 13, 2020 [30]. In the latter three segments, the role of vaccination gradually became dominant—surpassing the effect of epidemic-related behaviors on DIR. According to previous study, the effectiveness of the vaccine in the United States could reach 70–90% (within one month of vaccination) [41, 42]. It is worthy of attention that the comparison among the regression models results under these three segments for the speed of the epidemic spread has been constantly changing with the mutant strain [43, 44]. Compared with the fifth segment dominated by the delta strain, the vaccine was more effective in controlling the epidemic in the fourth segment. This may be the rapid increase in vaccine coverage from 0 to around 30–40% in these three months [30], targeted reductions in epidemic dominated by non-VOCs. In our model, AVD had a significant effect on DIR which was far exceeding the effect of epidemic-related behaviors while they still had significant contributions to reducing DIR.

The fifth segment model showed a decline in the impact of AVD. On the one hand, it might due to the effectiveness of the vaccine gradually decreases, and even dropped to 47% after five months [42]. On the other hand, the susceptibility to vaccines of the gradual dominance of the epidemic—the Delta strain had decreased [45, 46]. At this period, the effect of travel on DIR had risen again, but still lower than that of AVD. The last stage was when the epidemic dominated by Omicron, while the government gradually canceling control policies making long-distance travel became easier and more frequent [47]. This corresponded to our results that TR (>250 miles) played a huge role in the development of DIR during this period, while vaccine inhibition of DIR was relatively more pronounced, even reaching the highest coefficient in the second category model (6.44E+00).

After considering regression models for different cluster groups, we found that the third category model were the closest to the overall model. 7 states in the second category often came noteworthy results. They were more vulnerable to vaccination effect in the later period of the epidemic. Specifically in the fourth and sixth segments, the sensitivity of DIR to the vaccine even masked the effect of AHR and TR. However, the states in the first category showed different results from the overall model in the final segment, the effect of vaccine was significantly lower than that of TR (>250 miles)—it was also the exact opposite of the second category. It was an interesting phenomenon which was completely unavailable only from the total model. Different states’ circumstances could really make the effect of various factors vary.

Based on the results for different incubation periods shown in the S1–S3 Tables, the 7-day incubation period model was indeed robust overall in most periods. In addition, we also found that the coefficient for AVD in the 7-day model was higher than 3-day model, while 3-day higher than 10-day in the last segment, supporting the view from Dr. Post [35]. This might be related to the reduced incubation period of the Omicron strain—previous study suggest that the lag effect was about half of that of the original strain [48]. However, our research did not fully support this view, not only model fitting degree of the 7-day model was higher than that of 3-day model, but the coefficient of AVD under the 7-day model was basically higher than that from 3-day model.

As for the research results of weather factors, it continued to maintain its relatively controversial characteristics [20]. Unsegmented results whether at 3, 7 or 10 days of incubation, suggested a negative effect of T, H, WS, AP, PPTN on DIR, even though the association was very weak. But the segmented results showed different phenomenon. In the first and second segments—corresponding to March to July, 2020—from the low temperature in winter to the high temperature in summer, T acted positively on DIR. According to the early researches, the rise in temperature showed a positive effect on the incidence, reached its peak at 60.8–82.4°F [16]. Our results might fit this characteristic for U.S. is located in the northern hemisphere—most states have average temperature lower than 82.4°F during the first and second segments (Table 1). However, excessive temperature in summer might inhibit the spread of the virus to a certain degree, corresponding the third segment (mainly included the hottest summer and autumn) model results—T and DIR were negatively correlated.

Our research had not yet found the relationship between humidity and DIR in main model, even if it showed slight significant in some other models, the coefficient was too small which could be ignored. WS had a certain negative effect on DIR, which might be explained by the fact that the circulating air would take away the virus entrenched in one place, diluting the density and reducing the transmission power. Rainfall might also have a similar effect, especially in the late stage of the epidemic dominated by variant strains, the negative correlation coefficients of WS, PPTN and DIR in the fifth and sixth segments even increased to a certain extent compared with the main unsegment model.

Overall, compared with daily epidemic-related behaviors and vaccination, the effect of weather on DIR was not of an order of magnitude, but as a controversial factor, we still insisted on controlling the effect of weather indicators in the model, and the results might provide some support to the future researches. In addition, our study only focused on the dependent variable daily incidence, and the influencing factors considered also existed certain limitations. Therefore, we expect that more studies with dynamic effects appear to deeply explore the various factors affecting the development of the epidemic.

## Conclusion

Staying at home or getting vaccinated were particularly important inhibitive behaviors for the spread of COVID-19 in U.S, even when it’s in the period dominated by Omicron. Travel, especially long-distance interstate travel was a significant risk factor for the spread of epidemic. The spread also might be associated with weather, albeit to a lesser extent.

## Supporting information

S1 Table Multivariate analysis of influencing factors—IR postponed for 3, 7, 10 days, TR(>250 miles) as independent variable. (DOCX)

S2 Table Multivariate analysis of influencing factors—IR postponed for 3, 7, 10 days, TR(>25 miles) as independent variable. (DOCX)

S3 Table Multivariate analysis of influencing factors—IR postponed for 3, 7, 10 days, TR as independent variable. (DOCX)
