# Supplementary material for: A panel regression analysis for the COVID-19 epidemic in the United States
Source: PLoS One. 2022 Aug 19;17(8):e0273344. doi: 10.1371/journal.pone.0273344 (PMC9390909; doi:10.1371/journal.pone.0273344)
Supplement: S1 Table — (DOCX) [file pone.0273344.s001.docx]

**S1 Table. Multivariate analysis of influencing factors - IR postponed for 3, 7, 10 days, TR(＞250 miles) as independent variable**

| **Variables** | **IR postponed for 3 days** | | | | | | | | | | | | **IR postponed for 7 days** | | | | | | | | | | | | **IR postponed for 10 days** | | | | | | | | | | | |
| --- | --- | --- | --- | --- | --- | --- | --- | --- | --- | --- | --- | --- | --- | --- | --- | --- | --- | --- | --- | --- | --- | --- | --- | --- | --- | --- | --- | --- | --- | --- | --- | --- | --- | --- | --- | --- |
|  | **50 states** | | | **the first category** | | | **the second category** | | | **the third category** | | | **50 states** | | | **the first category** | | | **the second category** | | | **the third category** | | | **50 states** | | | **the first category** | | | **the second category** | | | **the third category** | | |
|  | **Coef.** | ***P*** | **R^2^** | **Coef.** | ***P*** | **R^2^** | **Coef.** | ***P*** | **R^2^** | **Coef.** | ***P*** | **R^2^** | **Coef.** | ***P*** | **R^2^** | **Coef.** | ***P*** | **R^2^** | **Coef.** | ***P*** | **R^2^** | **Coef.** | ***P*** | **R^2^** | **Coef.** | ***P*** | **R^2^** | **Coef.** | ***P*** | **R^2^** | **Coef.** | ***P*** | **R^2^** | **Coef.** | ***P*** | **R^2^** |
| **Unsegmented** |  |  |  |  |  |  |  |  |  |  |  |  |  |  |  |  |  |  |  |  |  |  |  |  |  |  |  |  |  |  |  |  |  |  |  |  |
| AHR | **-1.51E-01** | 0.000 | 0.34 | **-4.68E-02** | 0.006 | 0.24 | **-1.90E-01** | 0.000 | 0.46 | **-1.68E-01** | 0.000 | 0.36 | **-1.89E-01** | 0.000 | 0.44 | **-1.32E-01** | 0.000 | 0.36 | **-2.21E-01** | 0.000 | 0.55 | **-1.96E-01** | 0.000 | 0.45 | **-1.92E-01** | 0.000 | 0.42 | **-1.29E-01** | 0.000 | 0.35 | **-2.30E-01** | 0.000 | 0.53 | **-2.03E-01** | 0.000 | 0.43 |
| TR(＞250 miles) | **1.04E+00** | 0.000 |  | **8.23E-01** | 0.000 |  | **8.86E-01** | 0.000 |  | **9.98E-01** | 0.000 |  | **8.42E-01** | 0.000 |  | **1.24E+00** | 0.000 |  | **4.72E-01** | 0.001 |  | **7.80E-01** | 0.000 |  | **1.11E+00** | 0.000 |  | **1.48E+00** | 0.000 |  | **7.24E-01** | 0.000 |  | **1.02E+00** | 0.000 |  |
| AVD | **-1.81E+00** | 0.000 |  | **-2.41E+00** | 0.000 |  | **-2.68E+00** | 0.000 |  | **-1.64E+00** | 0.000 |  | **-2.29E+00** | 0.000 |  | **-2.59E+00** | 0.000 |  | **-3.05E+00** | 0.000 |  | **-2.18E+00** | 0.000 |  | **-2.23E+00** | 0.000 |  | **-2.49E+00** | 0.000 |  | **-3.10E+00** | 0.000 |  | **-2.14E+00** | 0.000 |  |
| T | **-4.84E-04** | 0.000 |  | **-4.41E-04** | 0.000 |  | **-4.90E-04** | 0.000 |  | **-4.94E-04** | 0.000 |  | **-5.02E-04** | 0.000 |  | **-5.57E-04** | 0.000 |  | **-5.67E-04** | 0.000 |  | **-4.99E-04** | 0.000 |  | **-4.86E-04** | 0.000 |  | **-5.40E-04** | 0.000 |  | **-5.62E-04** | 0.000 |  | **-4.90E-04** | 0.000 |  |
| H | -5.14E-06 | 0.549 |  | -2.47E-05 | 0.397 |  | **-6.39E-05** | 0.002 |  | **6.67E-05** | 0.000 |  | 9.80E-06 | 0.194 |  | 3.66E-05 | 0.141 |  | **-6.12E-05** | 0.002 |  | **4.14E-05** | 0.000 |  | **2.97E-05** | 0.000 |  | **9.27E-05** | 0.000 |  | **-5.56E-05** | 0.004 |  | **5.89E-05** | 0.000 |  |
| WS | **-2.52E-04** | 0.000 |  | **-7.10E-04** | 0.000 |  | **-8.49E-04** | 0.000 |  | 1.44E-05 | 0.781 |  | **-1.09E-04** | 0.005 |  | **-6.88E-04** | 0.000 |  | **-3.10E-04** | 0.000 |  | 4.17E-05 | 0.363 |  | **-9.22E-05** | 0.018 |  | **-6.58E-04** | 0.000 |  | **-1.67E-04** | 0.040 |  | -2.06E-05 | 0.651 |  |
| AP | **-2.27E-03** | 0.000 |  | **-2.04E-02** | 0.000 |  | **-2.98E-03** | 0.000 |  | -8.35E-04 | 0.075 |  | **-2.47E-03** | 0.000 |  | **-2.39E-02** | 0.000 |  | **-1.78E-03** | 0.000 |  | **-2.09E-03** | 0.000 |  | **-3.03E-03** | 0.000 |  | **-2.56E-02** | 0.000 |  | **-2.69E-03** | 0.000 |  | **-2.72E-03** | 0.000 |  |
| PPTN | **-2.57E-03** | 0.000 |  | 1.24E-03 | 0.473 |  | **-2.74E-02** | 0.000 |  | **-4.09E-03** | 0.000 |  | **-2.12E-03** | 0.000 |  | **-3.15E-03** | 0.033 |  | **-2.29E-02** | 0.000 |  | **-2.52E-03** | 0.000 |  | **-2.24E-03** | 0.000 |  | **-4.55E-03** | 0.002 |  | **-2.16E-02** | 0.000 |  | **-2.55E-03** | 0.000 |  |
| constant | 1.46E-01 | 0.000 |  | 6.69E-01 | 0.000 |  | 1.77E-01 | 0.000 |  | 1.03E-01 | 0.000 |  | 1.64E-01 | 0.000 |  | 7.89E-01 | 0.000 |  | 1.59E-01 | 0.000 |  | 1.53E-01 | 0.000 |  | 1.76E-01 | 0.000 |  | 8.32E-01 | 0.000 |  | 1.80E-01 | 0.000 |  | 1.69E-01 | 0.000 |  |
| **Segmented** |  |  |  |  |  |  |  |  |  |  |  |  |  |  |  |  |  |  |  |  |  |  |  |  |  |  |  |  |  |  |  |  |  |  |  |  |
| **Ⅰ** |  |  |  |  |  |  |  |  |  |  |  |  |  |  |  |  |  |  |  |  |  |  |  |  |  |  |  |  |  |  |  |  |  |  |  |  |
| AHR | **-4.88E-02** | 0.000 | 0.70 | **-5.63E-02** | 0.000 | 0.67 | **-4.07E-02** | 0.000 | 0.80 | **-4.94E-02** | 0.000 | 0.71 | **-5.25E-02** | 0.000 | 0.83 | **-5.77E-02** | 0.000 | 0.89 | **-7.51E-02** | 0.000 | 0.86 | **-4.73E-02** | 0.000 | 0.83 | **-5.28E-02** | 0.000 | 0.54 | **-6.14E-02** | 0.000 | 0.87 | **-6.74E-02** | 0.000 | 0.84 | -4.73E-02 | 0.000 | 0.81 |
| TR(＞250 miles) | **-7.92E-03** | 0.000 |  | -4.50E-03 | 0.372 |  | **-8.21E-03** | 0.000 |  | **-6.76E-03** | 0.000 |  | **-8.97E-03** | 0.000 |  | -3.46E-03 | 0.182 |  | -2.73E-02 | 0.377 |  | **-8.24E-03** | 0.000 |  | **-7.30E-03** | 0.000 |  | **-6.97E-03** | 0.006 |  | -2.76E-03 | 0.266 |  | **-7.44E-03** | 0.000 |  |
| T | 2.64E-06 | 0.843 |  | -1.56E-05 | 0.784 |  | -2.14E-05 | 0.393 |  | -2.27E-05 | 0.172 |  | **2.99E-05** | 0.003 |  | 4.75E-05 | 0.107 |  | -4.90E-05 | 0.188 |  | **7.30E-05** | 0.000 |  | **3.61E-05** | 0.000 |  | **6.76E-05** | 0.017 |  | **-6.91E-05** | 0.047 |  | 7.08E-05 | 0.000 |  |
| H | **1.95E-05** | 0.002 |  | -1.15E-05 | 0.641 |  | -9.22E-06 | 0.369 |  | **3.78E-05** | 0.000 |  | **-9.64E-06** | 0.045 |  | **-5.07E-05** | 0.000 |  | **-4.67E-05** | 0.002 |  | **1.46E-05** | 0.012 |  | 8.03E-06 | 0.079 |  | -2.23E-05 | 0.067 |  | -1.39E-05 | 0.328 |  | 2.52E-05 | 0.000 |  |
| WS | **1.24E-04** | 0.000 |  | 1.98E-04 | 0.174 |  | 7.21E-05 | 0.229 |  | **1.58E-04** | 0.000 |  | 2.91E-05 | 0.244 |  | **-2.25E-04** | 0.003 |  | **2.85E-04** | 0.002 |  | **6.21E-05** | 0.019 |  | 1.93E-05 | 0.414 |  | **-2.04E-04** | 0.005 |  | **2.10E-04** | 0.012 |  | **4.24E-05** | 0.087 |  |
| AP | **-1.79E-03** | 0.000 |  | **-8.62E-03** | 0.001 |  | -1.47E-03 | 0.266 |  | **-1.85E-03** | 0.000 |  | **-1.53E-03** | 0.000 |  | **-9.30E-03** | 0.000 |  | 1.08E-03 | 0.592 |  | **-9.50E-04** | 0.002 |  | **-6.56E-04** | 0.015 |  | **-4.48E-03** | 0.001 |  | -3.34E-04 | 0.855 |  | -3.65E-05 | 0.892 |  |
| PPTN | **-2.07E-03** | 0.000 |  | **-4.62E-03** | 0.036 |  | **-5.27E-03** | 0.021 |  | **-2.37E-03** | 0.000 |  | **-2.09E-03** | 0.000 |  | 2.14E-03 | 0.058 |  | -4.31E-03 | 0.198 |  | **-2.24E-03** | 0.000 |  | **-2.27E-03** | 0.000 |  | 1.74E-03 | 0.109 |  | **-7.61E-03** | 0.016 |  | **-2.23E-03** | 0.000 |  |
| constant | 9.23E-02 | 0.000 |  | 3.06E-01 | 0.000 |  | 7.36E-02 | 0.026 |  | 9.48E-02 | 0.000 |  | 8.99E-02 | 0.000 |  | 3.29E-01 | 0.000 |  | 3.60E-02 | 0.473 |  | 6.55E-02 | 0.000 |  | 6.39E-02 | 0.000 |  | 1.88E-01 | 0.000 |  | 6.71E-02 | 0.141 |  | 3.87E-02 | 0.000 |  |
| **Ⅱ** |  |  |  |  |  |  |  |  |  |  |  |  |  |  |  |  |  |  |  |  |  |  |  |  |  |  |  |  |  |  |  |  |  |  |  |  |
| AHR | **-1.45E-02** | 0.000 | 0.14 | **-4.70E-02** | 0.000 | 0.64 | **-3.35E-02** | 0.000 | 0.31 | -4.21E-03 | 0.137 | 0.10 | **-2.72E-02** | 0.000 | 0.18 | **-3.73E-02** | 0.000 | 0.30 | **-3.26E-02** | 0.000 | 0.20 | **-2.35E-02** | 0.000 | 0.18 | **-2.91E-02** | 0.000 | 0.20 | **-4.11E-02** | 0.000 | 0.31 | **-3.29E-02** | 0.000 | 0.23 | **-2.68E-02** | 0.000 | 0.20 |
| TR(＞250 miles) | **3.39E-01** | 0.000 |  | **6.73E-01** | 0.000 |  | **4.14E-01** | 0.000 |  | **2.12E-01** | 0.000 |  | **1.91E-01** | 0.000 |  | **2.04E-01** | 0.003 |  | **2.62E-01** | 0.000 |  | **1.66E-01** | 0.000 |  | **1.96E-01** | 0.000 |  | **1.74E-01** | 0.016 |  | **3.03E-01** | 0.000 |  | **1.64E-01** | 0.000 |  |
| T | **3.10E-05** | 0.000 |  | **4.63E-04** | 0.000 |  | **1.43E-04** | 0.000 |  | -9.82E-06 | 0.210 |  | **6.72E-05** | 0.000 |  | **1.34E-04** | 0.000 |  | **6.73E-05** | 0.000 |  | **5.78E-05** | 0.000 |  | **8.11E-05** | 0.000 |  | **1.70E-04** | 0.000 |  | **7.39E-05** | 0.000 |  | **7.39E-05** | 0.000 |  |
| H | **-1.40E-05** | 0.022 |  | 6.25E-06 | 0.769 |  | **4.72E-05** | 0.005 |  | -7.22E-06 | 0.299 |  | **1.33E-05** | 0.000 |  | **5.15E-05** | 0.000 |  | -1.11E-05 | 0.435 |  | **1.66E-05** | 0.000 |  | **7.62E-06** | 0.040 |  | **4.44E-05** | 0.003 |  | -2.31E-05 | 0.104 |  | **1.23E-05** | 0.004 |  |
| WS | **2.37E-04** | 0.000 |  | **2.05E-04** | 0.041 |  | -1.32E-04 | 0.054 |  | **2.14E-04** | 0.000 |  | **-7.78E-05** | 0.000 |  | -2.50E-05 | 0.710 |  | -6.64E-05 | 0.252 |  | **-9.19E-05** | 0.000 |  | **-1.01E-04** | 0.000 |  | -3.13E-05 | 0.655 |  | -7.60E-05 | 0.191 |  | **-1.15E-04** | 0.000 |  |
| AP | -2.46E-04 | 0.054 |  | **9.46E-03** | 0.000 |  | **-3.80E-04** | 0.003 |  | **-2.59E-03** | 0.000 |  | **-2.24E-04** | 0.003 |  | **4.43E-03** | 0.003 |  | -2.02E-04 | 0.062 |  | **-7.65E-04** | 0.008 |  | **-2.09E-04** | 0.007 |  | **6.20E-03** | 0.000 |  | -1.95E-04 | 0.072 |  | -4.98E-04 | 0.091 |  |
| PPTN | **2.89E-03** | 0.000 |  | **1.79E-03** | 0.013 |  | 2.36E-03 | 0.334 |  | **2.45E-03** | 0.000 |  | 3.30E-04 | 0.136 |  | 5.44E-04 | 0.263 |  | 9.53E-04 | 0.644 |  | 5.33E-05 | 0.838 |  | **5.31E-04** | 0.020 |  | 8.82E-04 | 0.081 |  | 3.15E-03 | 0.127 |  | 2.71E-04 | 0.313 |  |
| constant | 2.04E-02 | 0.000 |  | -2.73E-01 | 0.000 |  | 2.87E-02 | 0.000 |  | 8.49E-02 | 0.000 |  | 3.01E-02 | 0.000 |  | -1.06E-01 | 0.017 |  | 3.29E-02 | 0.000 |  | 4.39E-02 | 0.000 |  | 3.08E-02 | 0.000 |  | -1.57E-01 | 0.001 |  | 3.29E-02 | 0.000 |  | 3.82E-02 | 0.000 |  |
| **Ⅲ** |  |  |  |  |  |  |  |  |  |  |  |  |  |  |  |  |  |  |  |  |  |  |  |  |  |  |  |  |  |  |  |  |  |  |  |  |
| AHR | **-1.79E-01** | 0.000 | 0.72 | **-1.73E-01** | 0.000 | 0.56 | **-2.24E-01** | 0.000 | 0.69 | **-1.63E-01** | 0.000 | 0.72 | **-1.80E-01** | 0.000 | 0.78 | **-1.88E-01** | 0.000 | 0.80 | **-1.53E-01** | 0.000 | 0.83 | **-1.85E-01** | 0.000 | 0.77 | **-1.55E-01** | 0.000 | 0.76 | **-1.77E-01** | 0.000 | 0.80 | **-1.24E-01** | 0.000 | 0.81 | **-1.60E-01** | 0.000 | 0.78 |
| TR(＞250 miles) | 7.20E-03 | 0.536 |  | 4.98E-02 | 0.156 |  | **-1.65E-02** | 0.003 |  | **2.90E-02** | 0.027 |  | **6.80E-03** | 0.000 |  | 8.42E-05 | 0.987 |  | **1.39E-02** | 0.002 |  | **7.24E-03** | 0.000 |  | **-2.96E-02** | 0.000 |  | **-2.11E-02** | 0.000 |  | **-2.75E-02** | 0.000 |  | **-3.03E-02** | 0.000 |  |
| T | **-2.12E-04** | 0.000 |  | **-1.13E-04** | 0.000 |  | **-2.46E-04** | 0.000 |  | **-2.28E-04** | 0.000 |  | **-1.95E-04** | 0.000 |  | **-2.04E-04** | 0.000 |  | **-1.81E-04** | 0.000 |  | **-1.90E-04** | 0.000 |  | **-2.16E-04** | 0.000 |  | **-2.23E-04** | 0.000 |  | **-2.18E-04** | 0.000 |  | **-2.11E-04** | 0.000 |  |
| H | **-3.89E-05** | 0.000 |  | **1.36E-04** | 0.000 |  | **-7.20E-05** | 0.000 |  | **-5.25E-05** | 0.000 |  | **-1.62E-05** | 0.000 |  | **3.93E-05** | 0.010 |  | **3.69E-05** | 0.001 |  | **-1.39E-05** | 0.015 |  | **-2.63E-05** | 0.000 |  | 2.04E-05 | 0.165 |  | -4.39E-07 | 0.968 |  | **-1.79E-05** | 0.002 |  |
| WS | **2.35E-04** | 0.000 |  | **-4.41E-04** | 0.000 |  | **3.50E-04** | 0.000 |  | **3.01E-04** | 0.000 |  | 2.01E-05 | 0.301 |  | **-1.57E-04** | 0.016 |  | 5.10E-05 | 0.267 |  | **4.66E-05** | 0.044 |  | -4.63E-06 | 0.810 |  | **-1.31E-04** | 0.037 |  | 2.33E-05 | 0.610 |  | 1.67E-05 | 0.469 |  |
| AP | 1.71E-05 | 0.883 |  | 5.87E-04 | 0.624 |  | **2.36E-03** | 0.000 |  | -1.31E-04 | 0.299 |  | **1.00E-03** | 0.000 |  | **3.48E-03** | 0.000 |  | **1.36E-03** | 0.000 |  | **8.90E-04** | 0.000 |  | **9.77E-04** | 0.000 |  | **3.20E-03** | 0.000 |  | **1.01E-03** | 0.000 |  | **8.87E-04** | 0.000 |  |
| PPTN | -2.58E-04 | 0.418 |  | -4.32E-04 | 0.514 |  | **-1.71E-02** | 0.000 |  | 4.83E-05 | 0.895 |  | **7.03E-04** | 0.006 |  | -1.01E-03 | 0.052 |  | **-2.91E-02** | 0.000 |  | **1.37E-03** | 0.000 |  | **8.28E-04** | 0.001 |  | **-9.78E-04** | 0.049 |  | **-2.54E-02** | 0.000 |  | **1.39E-03** | 0.000 |  |
| constant | 1.62E-01 | 0.000 |  | 1.36E-01 | 0.001 |  | 1.41E-01 | 0.000 |  | 1.55E-01 | 0.000 |  | 1.33E-01 | 0.000 |  | 7.29E-02 | 0.019 |  | 9.91E-02 | 0.000 |  | 1.38E-01 | 0.000 |  | 1.27E-01 | 0.000 |  | 8.06E-02 | 0.007 |  | 1.02E-01 | 0.000 |  | 1.32E-01 | 0.000 |  |
| **Ⅳ** |  |  |  |  |  |  |  |  |  |  |  |  |  |  |  |  |  |  |  |  |  |  |  |  |  |  |  |  |  |  |  |  |  |  |  |  |
| AHR | **9.05E-02** | 0.000 | 0.59 | **-7.77E-02** | 0.000 | 0.63 | **-4.63E-02** | 0.000 | 0.61 | **-7.75E-02** | 0.000 | 0.55 | **-5.87E-02** | 0.000 | 0.69 | **-2.73E-02** | 0.026 | 0.73 | -1.31E-02 | 0.164 | 0.72 | **-6.87E-02** | 0.000 | 0.70 | **-7.30E-02** | 0.000 | 0.48 | -6.20E-03 | 0.594 | 0.53 | **-2.85E-02** | 0.002 | 0.40 | **-8.08E-02** | 0.000 | 0.52 |
| TR(＞250 miles) | **-3.01E-02** | 0.000 |  | -1.79E-01 | 0.170 |  | **1.52E-01** | 0.017 |  | -5.29E-02 | 0.275 |  | -5.01E-02 | 0.131 |  | 1.14E-01 | 0.255 |  | -3.02E-02 | 0.689 |  | -6.66E-02 | 0.087 |  | **1.55E-01** | 0.000 |  | **9.77E-02** | 0.300 |  | **2.13E-01** | 0.003 |  | **1.64E-01** | 0.000 |  |
| AVD | **-5.69E-01** | 0.000 |  | **-3.71E+00** | 0.000 |  | **-1.02E+00** | 0.000 |  | **-6.31E-01** | 0.000 |  | **-1.05E+00** | 0.000 |  | **-2.35E+00** | 0.000 |  | **-1.51E+00** | 0.000 |  | **-8.99E-01** | 0.000 |  | **-9.18E-01** | 0.000 |  | **-2.08E+00** | 0.000 |  | **-1.35E+00** | 0.000 |  | **-7.86E-01** | 0.000 |  |
| T | **1.47E-04** | 0.000 |  | 2.42E-06 | 0.930 |  | **1.01E-04** | 0.000 |  | **1.13E-04** | 0.000 |  | **1.38E-04** | 0.000 |  | **6.66E-05** | 0.002 |  | **5.23E-05** | 0.006 |  | **1.42E-04** | 0.000 |  | **1.57E-04** | 0.000 |  | **1.20E-04** | 0.000 |  | **6.47E-05** | 0.000 |  | **1.62E-04** | 0.000 |  |
| H | **-2.16E-05** | 0.002 |  | **5.55E-05** | 0.016 |  | **1.04E-04** | 0.000 |  | **-2.06E-05** | 0.009 |  | **1.16E-05** | 0.033 |  | **-3.55E-05** | 0.045 |  | 1.46E-05 | 0.349 |  | **1.96E-05** | 0.002 |  | -8.38E-06 | 0.106 |  | **-6.47E-05** | 0.000 |  | -2.36E-06 | 0.874 |  | -2.31E-06 | 0.702 |  |
| WS | **-3.90E-04** | 0.000 |  | **-6.03E-04** | 0.000 |  | **-1.10E-04** | 0.021 |  | **-4.67E-04** | 0.000 |  | **-1.89E-04** | 0.000 |  | **-5.40E-04** | 0.000 |  | -1.08E-05 | 0.847 |  | **-2.05E-04** | 0.000 |  | **-1.61E-04** | 0.000 |  | **-4.01E-04** | 0.000 |  | -1.42E-05 | 0.790 |  | **-1.71E-04** | 0.000 |  |
| AP | -4.12E-04 | 0.339 |  | **-4.98E-03** | 0.021 |  | **1.02E-02** | 0.000 |  | **-2.82E-03** | 0.000 |  | **-9.62E-04** | 0.006 |  | **-7.19E-03** | 0.000 |  | **5.97E-03** | 0.000 |  | **-1.55E-03** | 0.000 |  | **-9.80E-04** | 0.003 |  | **-6.49E-03** | 0.000 |  | **4.73E-03** | 0.000 |  | **-1.47E-03** | 0.000 |  |
| PPTN | **-8.78E-04** | 0.043 |  | **-6.35E-03** | 0.000 |  | **-1.42E-02** | 0.000 |  | -7.63E-04 | 0.090 |  | **-1.81E-03** | 0.000 |  | **-5.59E-03** | 0.000 |  | -3.62E-03 | 0.376 |  | **-1.61E-03** | 0.000 |  | **-8.41E-04** | 0.011 |  | **-4.04E-03** | 0.001 |  | -3.54E-03 | 0.364 |  | -6.49E-04 | 0.060 |  |
| constant | 1.92E-02 | 0.129 |  | 2.03E-01 | 0.002 |  | -2.08E-01 | 0.000 |  | 1.65E-01 | 0.000 |  | 9.29E-02 | 0.000 |  | 2.52E-01 | 0.000 |  | -1.15E-01 | 0.000 |  | 1.17E-01 | 0.000 |  | 1.02E-01 | 0.000 |  | 2.28E-01 | 0.000 |  | -7.49E-02 | 0.013 |  | 1.22E-01 | 0.000 |  |
| **Ⅴ** |  |  |  |  |  |  |  |  |  |  |  |  |  |  |  |  |  |  |  |  |  |  |  |  |  |  |  |  |  |  |  |  |  |  |  |  |
| AHR | **-2.09E-02** | 0.003 | 0.19 | **-7.25E-02** | 0.000 | 0.41 | **-7.65E-02** | 0.000 | 0.30 | **-4.46E-02** | 0.000 | 0.19 | **-4.51E-02** | 0.000 | 0.18 | **-6.87E-02** | 0.000 | 0.51 | **-1.78E-01** | 0.000 | 0.28 | **-4.48E-02** | 0.000 | 0.19 | **-6.35E-02** | 0.000 | 0.19 | **-6.22E-02** | 0.000 | 0.41 | **-2.10E-01** | 0.000 | 0.23 | **-6.48E-02** | 0.000 | 0.21 |
| TR(＞250 miles) | **9.08E-01** | 0.000 |  | **1.34E+00** | 0.000 |  | **8.41E-01** | 0.000 |  | **3.10E-01** | 0.000 |  | **2.26E-01** | 0.000 |  | **7.65E-01** | 0.000 |  | **2.91E-01** | 0.028 |  | 1.86E-02 | 0.770 |  | **2.89E-01** | 0.000 |  | **9.40E-01** | 0.000 |  | **2.97E-01** | 0.023 |  | 6.79E-02 | 0.284 |  |
| AVD | **-1.05E-05** | 0.000 |  | **-2.73E-05** | 0.000 |  | **-9.84E-01** | 0.000 |  | -8.78E-03 | 0.819 |  | **-5.44E-01** | 0.000 |  | **-1.73E-05** | 0.000 |  | **-8.79E-01** | 0.000 |  | **-6.70E-01** | 0.000 |  | **-7.06E-01** | 0.000 |  | **-1.55E-05** | 0.000 |  | **-1.06E+00** | 0.000 |  | **-8.37E-01** | 0.000 |  |
| T | **-1.00E-04** | 0.000 |  | **3.81E-04** | 0.000 |  | **-3.70E-04** | 0.000 |  | **-2.80E-04** | 0.000 |  | **-2.32E-04** | 0.000 |  | **-1.16E-04** | 0.000 |  | **-2.40E-04** | 0.000 |  | **-2.67E-04** | 0.000 |  | **-2.37E-04** | 0.000 |  | **-1.10E-04** | 0.000 |  | **-2.32E-04** | 0.000 |  | **-2.76E-04** | 0.000 |  |
| H | **1.35E-04** | 0.000 |  | **1.41E-04** | 0.000 |  | **-1.40E-04** | 0.000 |  | **1.61E-04** | 0.000 |  | **7.59E-05** | 0.000 |  | **2.09E-04** | 0.000 |  | **-3.78E-05** | 0.036 |  | **1.06E-04** | 0.000 |  | **7.04E-05** | 0.000 |  | **2.06E-04** | 0.000 |  | **-2.91E-05** | 0.102 |  | **9.65E-05** | 0.000 |  |
| WS | **-9.19E-04** | 0.000 |  | **-1.97E-03** | 0.000 |  | **-8.96E-04** | 0.000 |  | **-6.52E-04** | 0.000 |  | **-7.08E-04** | 0.000 |  | **-1.35E-03** | 0.000 |  | **-7.90E-04** | 0.000 |  | **-5.77E-04** | 0.000 |  | **-7.47E-04** | 0.000 |  | **-1.41E-03** | 0.000 |  | **-8.10E-04** | 0.000 |  | **-6.28E-04** | 0.000 |  |
| AP | **-4.98E-03** | 0.000 |  | **5.86E-03** | 0.002 |  | **-1.81E-03** | 0.001 |  | **-7.35E-03** | 0.000 |  | **-3.29E-03** | 0.000 |  | **-9.53E-03** | 0.000 |  | -5.95E-04 | 0.216 |  | **-5.08E-03** | 0.000 |  | **-3.40E-03** | 0.000 |  | **-8.24E-03** | 0.000 |  | -6.23E-04 | 0.190 |  | **-5.51E-03** | 0.000 |  |
| PPTN | **-2.02E-03** | 0.000 |  | **2.11E-03** | 0.017 |  | **-8.82E-03** | 0.008 |  | **-3.53E-03** | 0.000 |  | **-2.54E-03** | 0.000 |  | **-4.33E-03** | 0.000 |  | **-9.49E-03** | 0.001 |  | **-3.04E-03** | 0.000 |  | **-2.34E-03** | 0.000 |  | **-3.95E-03** | 0.000 |  | **-1.07E-02** | 0.000 |  | **-2.72E-03** | 0.000 |  |
| constant | 1.77E-01 | 0.000 |  | -1.25E-01 | 0.031 |  | 1.13E-01 | 0.000 |  | 2.76E-01 | 0.000 |  | 1.37E-01 | 0.000 |  | 3.56E-01 | 0.000 |  | 9.51E-02 | 0.000 |  | 1.92E-01 | 0.000 |  | 1.45E-01 | 0.000 |  | 3.11E-01 | 0.000 |  | 1.03E-01 | 0.000 |  | 2.11E-01 | 0.000 |  |
| **Ⅵ** |  |  |  |  |  |  |  |  |  |  |  |  |  |  |  |  |  |  |  |  |  |  |  |  |  |  |  |  |  |  |  |  |  |  |  |  |
| AHR | **-3.02E-01** | 0.000 | 0.13 | -1.36E-01 | 0.492 | 0.16 | -7.95E-02 | 0.487 | 0.21 | -2.01E-01 | 0.000 | 0.18 | **-1.66E-01** | 0.000 | 0.21 | 9.56E-02 | 0.581 | 0.11 | **-5.75E-01** | 0.000 | 0.34 | -6.73E-02 | 0.133 | 0.27 | **-1.42E-01** | 0.001 | 0.15 | 1.02E-02 | 0.952 | 0.09 | **-8.40E-01** | 0.000 | 0.30 | **-1.80E-01** | 0.000 | 0.20 |
| TR(＞250 miles) | **2.45E+00** | 0.000 |  | 3.07E+00 | 0.083 |  | 2.39E-01 | 0.835 |  | **3.16E+00** | 0.000 |  | **2.26E+00** | 0.000 |  | **3.63E+00** | 0.019 |  | 1.50E-01 | 0.888 |  | **3.48E+00** | 0.000 |  | **3.95E+00** | 0.000 |  | **4.51E+00** | 0.003 |  | 1.13E+00 | 0.290 |  | **4.40E+00** | 0.000 |  |
| AVD | **-1.05E+00** | 0.000 |  | -2.56E+00 | 0.118 |  | **-8.06E+00** | 0.000 |  | **-7.58E-01** | 0.004 |  | **-2.32E+00** | 0.000 |  | -1.90E+00 | 0.184 |  | **-6.44E+00** | 0.000 |  | **-2.13E+00** | 0.000 |  | **-9.55E-01** | 0.000 |  | -9.18E-01 | 0.518 |  | **-5.65E+00** | 0.000 |  | **-9.98E-01** | 0.000 |  |
| T | **-5.39E-04** | 0.000 |  | -1.33E-04 | 0.416 |  | **9.61E-04** | 0.000 |  | **-6.05E-04** | 0.000 |  | **-6.63E-04** | 0.000 |  | -4.36E-04 | 0.002 |  | **4.82E-04** | 0.000 |  | **-7.27E-04** | 0.000 |  | **-6.02E-04** | 0.000 |  | -3.88E-04 | 0.006 |  | **3.74E-04** | 0.004 |  | **-6.48E-04** | 0.000 |  |
| H | **3.05E-04** | 0.000 |  | **-4.84E-04** | 0.000 |  | **5.71E-04** | 0.000 |  | **4.73E-04** | 0.000 |  | **3.09E-04** | 0.000 |  | 1.91E-05 | 0.871 |  | **5.40E-04** | 0.000 |  | **3.82E-04** | 0.000 |  | **4.47E-04** | 0.000 |  | 2.15E-04 | 0.066 |  | **4.47E-04** | 0.000 |  | **5.05E-04** | 0.000 |  |
| WS | 4.07E-05 | 0.808 |  | 1.22E-03 | 0.133 |  | **-2.80E-03** | 0.000 |  | **1.01E-03** | 0.000 |  | 2.57E-04 | 0.071 |  | 7.56E-04 | 0.286 |  | **-1.91E-03** | 0.000 |  | **9.36E-04** | 0.000 |  | **4.22E-04** | 0.004 |  | 6.13E-04 | 0.382 |  | **-1.43E-03** | 0.000 |  | **8.93E-04** | 0.000 |  |
| AP | **1.89E-03** | 0.008 |  | -1.16E-02 | 0.112 |  | **-1.10E-02** | 0.000 |  | **6.61E-03** | 0.000 |  | **1.62E-03** | 0.007 |  | **-7.11E-03** | 0.266 |  | **-8.83E-03** | 0.000 |  | **5.68E-03** | 0.000 |  | 4.34E-04 | 0.483 |  | **-1.29E-02** | 0.042 |  | **-9.78E-03** | 0.000 |  | **4.85E-03** | 0.000 |  |
| PPTN | **-1.72E-02** | 0.000 |  | 3.39E-03 | 0.700 |  | **-7.14E-02** | 0.001 |  | **-1.85E-02** | 0.000 |  | **-9.44E-03** | 0.000 |  | -6.48E-03 | 0.400 |  | -3.96E-02 | 0.050 |  | **-9.11E-03** | 0.000 |  | **-9.98E-03** | 0.000 |  | **-1.82E-02** | 0.017 |  | -1.07E-02 | 0.598 |  | **-8.54E-03** | 0.001 |  |
| constant | 2.09E-01 | 0.000 |  | 5.03E-01 | 0.012 |  | 3.16E-01 | 0.000 |  | -3.50E-02 | 0.525 |  | 2.17E-02 | 0.261 |  | 2.22E-01 | 0.305 |  | 3.75E-01 | 0.000 |  | -1.39E-01 | 0.000 |  | 1.90E-02 | 0.338 |  | 3.87E-01 | 0.072 |  | 4.49E-01 | 0.000 |  | -1.13E-01 | 0.000 |  |

AHR, the proportion of daily residents at home; TR, Daily trips per capita; AVD, daily administered vaccination dose per capita; T, temperature; H, humidity; WS, wind speed; AP, air pressure; PPTN, precipitation
