# Supplementary material for: A panel regression analysis for the COVID-19 epidemic in the United States
Source: PLoS One. 2022 Aug 19;17(8):e0273344. doi: 10.1371/journal.pone.0273344 (PMC9390909; doi:10.1371/journal.pone.0273344)
Supplement: S2 Table — (DOCX) [file pone.0273344.s002.docx]

**S2 Table. Multivariate analysis of influencing factors - IR postponed for 3, 7, 10 days, TR(＞25 miles) as independent variable**

| **Variables** | **IR postponed for 3 days** | | | | | | | | | | | | **IR postponed for 7 days** | | | | | | | | | | | | **IR postponed for 10 days** | | | | | | | | | | | |
| --- | --- | --- | --- | --- | --- | --- | --- | --- | --- | --- | --- | --- | --- | --- | --- | --- | --- | --- | --- | --- | --- | --- | --- | --- | --- | --- | --- | --- | --- | --- | --- | --- | --- | --- | --- | --- |
|  | **50 states** | | | **the first category** | | | **the second category** | | | **the third category** | | | **50 states** | | | **the first category** | | | **the second category** | | | **the third category** | | | **50 states** | | | **the first category** | | | **the second category** | | | **the third category** | | |
|  | **Coef.** | ***P*** | **R^2^** | **Coef.** | ***P*** | **R^2^** | **Coef.** | ***P*** | **R^2^** | **Coef.** | ***P*** | **R^2^** | **Coef.** | ***P*** | **R^2^** | **Coef.** | ***P*** | **R^2^** | **Coef.** | ***P*** | **R^2^** | **Coef.** | ***P*** | **R^2^** | **Coef.** | ***P*** | **R^2^** | **Coef.** | ***P*** | **R^2^** | **Coef.** | ***P*** | **R^2^** | **Coef.** | ***P*** | **R^2^** |
| **Unsegmented** |  |  |  |  |  |  |  |  |  |  |  |  |  |  |  |  |  |  |  |  |  |  |  |  |  |  |  |  |  |  |  |  |  |  |  |  |
| AHR | **-0.14989** | 0.000 | 0.33 | **-0.07013** | 0.000 | 0.23 | **-0.19663** | 0.000 | 0.45 | **-0.16236** | 0.000 | 0.36 | **-0.18291** | 0.000 | 0.44 | **-0.13597** | 0.000 | 0.34 | **-0.22328** | 0.000 | 0.53 | **-0.18830** | 0.000 | 0.44 | **-0.18967** | 0.000 | 0.41 | **-0.13469** | 0.000 | 0.33 | **-0.23631** | 0.000 | 0.52 | **-0.19955** | 0.000 | 0.43 |
| TR(＞25 miles) | **0.02388** | 0.000 |  | **-0.04552** | 0.000 |  | 0.00657 | 0.415 |  | **0.03101** | 0.000 |  | **0.03278** | 0.000 |  | **0.03130** | 0.003 |  | 0.00652 | 0.379 |  | **0.03457** | 0.000 |  | **0.02869** | 0.000 |  | **0.03706** | 0.000 |  | 0.00206 | 0.781 |  | **0.02693** | 0.000 |  |
| AVD | **-1.97632** | 0.000 |  | **-2.71128** | 0.000 |  | **-2.86335** | 0.000 |  | **-1.78619** | 0.000 |  | **-2.42142** | 0.000 |  | **-2.80848** | 0.000 |  | **-3.14342** | 0.000 |  | **-2.29180** | 0.000 |  | **-2.40720** | 0.000 |  | **-2.75996** | 0.000 |  | **-3.24583** | 0.000 |  | **-2.28743** | 0.000 |  |
| T | **-0.00049** | 0.000 |  | **-0.00041** | 0.000 |  | **-0.00049** | 0.000 |  | **-0.00050** | 0.000 |  | **-0.00051** | 0.000 |  | **-0.00057** | 0.000 |  | **-0.00057** | 0.000 |  | **-0.00051** | 0.000 |  | **-0.00049** | 0.000 |  | **-0.00056** | 0.000 |  | **-0.00056** | 0.000 |  | **-0.00050** | 0.000 |  |
| H | 0.00000 | 0.937 |  | -0.00003 | 0.234 |  | **-0.00007** | 0.001 |  | **0.00008** | 0.000 |  | **0.00002** | 0.034 |  | 0.00005 | 0.060 |  | **-0.00006** | 0.001 |  | **0.00005** | 0.000 |  | **0.00004** | 0.000 |  | **0.00011** | 0.000 |  | **-0.00006** | 0.002 |  | **0.00007** | 0.000 |  |
| WS | **-0.00028** | 0.000 |  | **-0.00085** | 0.000 |  | **-0.00088** | 0.000 |  | -0.00001 | 0.891 |  | **-0.00013** | 0.001 |  | **-0.00077** | 0.000 |  | **-0.00033** | 0.000 |  | 0.00002 | 0.599 |  | **-0.00012** | 0.002 |  | **-0.00076** | 0.000 |  | **-0.00019** | 0.021 |  | -0.00005 | 0.302 |  |
| AP | **-0.00230** | 0.000 |  | **-0.02092** | 0.000 |  | **-0.00303** | 0.000 |  | -0.00082 | 0.080 |  | **-0.00248** | 0.000 |  | **-0.02437** | 0.000 |  | **-0.00181** | 0.000 |  | **-0.00206** | 0.000 |  | **-0.00306** | 0.000 |  | **-0.02616** | 0.000 |  | **-0.00272** | 0.000 |  | **-0.00274** | 0.000 |  |
| PPTN | **-0.00272** | 0.000 |  | 0.00188 | 0.278 |  | **-0.02667** | 0.000 |  | **-0.00435** | 0.000 |  | **-0.00229** | 0.000 |  | **-0.00323** | 0.030 |  | **-0.02257** | 0.000 |  | **-0.00275** | 0.000 |  | **-0.00242** | 0.000 |  | **-0.00464** | 0.002 |  | **-0.02104** | 0.000 |  | **-0.00280** | 0.000 |  |
| constant | 0.14959 | 0.000 |  | 0.70932 | 0.000 |  | 0.18743 | 0.000 |  | 0.10104 | 0.000 |  | 0.16109 | 0.000 |  | 0.80841 | 0.000 |  | 0.16301 | 0.000 |  | 0.14684 | 0.000 |  | 0.17787 | 0.000 |  | 0.85480 | 0.000 |  | 0.18873 | 0.000 |  | 0.16950 | 0.000 |  |
| **Segmented** |  |  |  |  |  |  |  |  |  |  |  |  |  |  |  |  |  |  |  |  |  |  |  |  |  |  |  |  |  |  |  |  |  |  |  |  |
| **Ⅰ** |  |  |  |  |  |  |  |  |  |  |  |  |  |  |  |  |  |  |  |  |  |  |  |  |  |  |  |  |  |  |  |  |  |  |  |  |
| AHR | **-0.05465** | 0.000 | 0.70 | **-0.05950** | 0.000 | 0.68 | **-0.04535** | 0.000 | 0.84 | **-0.05405** | 0.000 | 0.72 | **-0.06005** | 0.000 | 0.81 | **-0.05928** | 0.000 | 0.90 | **-0.06834** | 0.000 | 0.87 | **-0.05411** | 0.000 | 0.82 | **-0.05889** | 0.000 | 0.80 | **-0.06572** | 0.000 | 0.90 | **-0.06847** | 0.000 | 0.85 | **-0.05338** | 0.000 | 0.81 |
| TR(＞25 miles) | **-0.07540** | 0.000 |  | -0.03056 | 0.596 |  | **-0.08012** | 0.000 |  | **-0.07653** | 0.000 |  | **-0.04555** | 0.000 |  | **-0.11059** | 0.000 |  | **-0.00812** | 0.002 |  | **-0.04511** | 0.000 |  | **-0.03923** | 0.000 |  | **-0.10731** | 0.000 |  | -0.03910 | 0.180 |  | **-0.04207** | 0.000 |  |
| T | 0.00000 | 0.940 |  | -0.00002 | 0.747 |  | -0.00005 | 0.059 |  | -0.00003 | 0.123 |  | **0.00003** | 0.007 |  | 0.00005 | 0.095 |  | -0.00003 | 0.361 |  | **0.00007** | 0.000 |  | **0.00003** | 0.000 |  | **0.00006** | 0.020 |  | **-0.00008** | 0.022 |  | **0.00007** | 0.000 |  |
| H | **0.00002** | 0.003 |  | -0.00001 | 0.566 |  | -0.00001 | 0.288 |  | **0.00004** | 0.000 |  | **-0.00001** | 0.003 |  | **-0.00004** | 0.001 |  | **-0.00003** | 0.024 |  | **0.00001** | 0.045 |  | 0.00000 | 0.360 |  | -0.00002 | 0.147 |  | -0.00001 | 0.406 |  | **0.00002** | 0.000 |  |
| WS | **0.00013** | 0.000 |  | 0.00019 | 0.200 |  | 0.00011 | 0.089 |  | **0.00016** | 0.000 |  | 0.00004 | 0.155 |  | **-0.00028** | 0.000 |  | **0.00029** | 0.001 |  | **0.00007** | 0.006 |  | 0.00003 | 0.299 |  | **-0.00025** | 0.001 |  | **0.00023** | 0.007 |  | **0.00005** | 0.044 |  |
| AP | **-0.00222** | 0.000 |  | **-0.00959** | 0.000 |  | **-0.00286** | 0.042 |  | **-0.00205** | 0.000 |  | **-0.00235** | 0.000 |  | **-0.00919** | 0.000 |  | 0.00146 | 0.439 |  | **-0.00167** | 0.000 |  | **-0.00131** | 0.000 |  | **-0.00540** | 0.000 |  | -0.00104 | 0.585 |  | **-0.00063** | 0.015 |  |
| PPTN | **-0.00225** | 0.000 |  | **-0.00464** | 0.036 |  | **-0.00595** | 0.011 |  | **-0.00253** | 0.000 |  | **-0.00221** | 0.000 |  | 0.00184 | 0.090 |  | -0.00438 | 0.178 |  | **-0.00239** | 0.000 |  | **-0.00237** | 0.000 |  | 0.00151 | 0.155 |  | **-0.00802** | 0.012 |  | **-0.00237** | 0.000 |  |
| constant | 0.10816 | 0.000 |  | 0.33741 | 0.000 |  | 0.11191 | 0.001 |  | 0.10321 | 0.000 |  | 0.11792 | 0.000 |  | 0.32691 | 0.000 |  | 0.02197 | 0.641 |  | 0.09037 | 0.000 |  | 0.08612 | 0.000 |  | 0.21758 | 0.000 |  | 0.08556 | 0.072 |  | 0.05932 | 0.000 |  |
| **Ⅱ** |  |  |  |  |  |  |  |  |  |  |  |  |  |  |  |  |  |  |  |  |  |  |  |  |  |  |  |  |  |  |  |  |  |  |  |  |
| AHR | **-0.03016** | 0.000 | 0.13 | **-0.06870** | 0.000 | 0.62 | **-0.05520** | 0.000 | 0.32 | **-0.01389** | 0.000 | 0.11 | **-0.03858** | 0.000 | 0.19 | **-0.05103** | 0.000 | 0.31 | **-0.04132** | 0.000 | 0.17 | **-0.03424** | 0.000 | 0.19 | **-0.03734** | 0.000 | 0.20 | **-0.04868** | 0.000 | 0.31 | **-0.04173** | 0.000 | 0.20 | **-0.03410** | 0.000 | 0.20 |
| TR(＞25 miles) | **0.01819** | 0.000 |  | **0.02274** | 0.000 |  | **0.02638** | 0.000 |  | **0.01153** | 0.000 |  | **0.01395** | 0.000 |  | **0.01686** | 0.000 |  | **0.00996** | 0.001 |  | **0.01386** | 0.000 |  | **0.00936** | 0.000 |  | **0.00869** | 0.013 |  | **0.00970** | 0.002 |  | **0.00858** | 0.000 |  |
| T | **0.00004** | 0.000 |  | **0.00049** | 0.000 |  | **0.00016** | 0.000 |  | -0.00001 | 0.494 |  | **0.00007** | 0.000 |  | **0.00012** | 0.000 |  | **0.00009** | 0.000 |  | **0.00005** | 0.000 |  | **0.00009** | 0.000 |  | **0.00017** | 0.000 |  | **0.00010** | 0.000 |  | **0.00008** | 0.000 |  |
| H | **-0.00001** | 0.032 |  | 0.00003 | 0.193 |  | **0.00006** | 0.000 |  | -0.00001 | 0.285 |  | **0.00001** | 0.001 |  | **0.00005** | 0.000 |  | 0.00000 | 0.862 |  | **0.00001** | 0.001 |  | **0.00001** | 0.020 |  | **0.00005** | 0.001 |  | -0.00001 | 0.376 |  | **0.00001** | 0.004 |  |
| WS | **0.00019** | 0.000 |  | 0.00008 | 0.441 |  | **-0.00015** | 0.025 |  | **0.00018** | 0.000 |  | **-0.00010** | 0.000 |  | -0.00006 | 0.352 |  | -0.00008 | 0.147 |  | **-0.00012** | 0.000 |  | **-0.00013** | 0.000 |  | -0.00006 | 0.356 |  | -0.00010 | 0.094 |  | **-0.00014** | 0.000 |  |
| AP | **-0.00026** | 0.042 |  | **0.00805** | 0.000 |  | **-0.00043** | 0.001 |  | **-0.00276** | 0.000 |  | **-0.00023** | 0.003 |  | **0.00347** | 0.018 |  | **-0.00025** | 0.024 |  | **-0.00095** | 0.001 |  | **-0.00022** | 0.005 |  | **0.00568** | 0.000 |  | **-0.00025** | 0.024 |  | **-0.00062** | 0.035 |  |
| PPTN | **0.00290** | 0.000 |  | **0.00225** | 0.002 |  | 0.00273 | 0.259 |  | **0.00235** | 0.000 |  | 0.00035 | 0.114 |  | 0.00059 | 0.212 |  | 0.00158 | 0.447 |  | 0.00001 | 0.958 |  | **0.00053** | 0.020 |  | 0.00098 | 0.052 |  | 0.00399 | 0.058 |  | 0.00019 | 0.468 |  |
| constant | 0.03044 | 0.000 |  | -0.21756 | 0.002 |  | 0.04122 | 0.000 |  | 0.09591 | 0.000 |  | 0.03705 | 0.000 |  | -0.06815 | 0.123 |  | 0.03864 | 0.000 |  | 0.05589 | 0.000 |  | 0.03621 | 0.000 |  | -0.13690 | 0.004 |  | 0.03892 | 0.000 |  | 0.04646 | 0.000 |  |
| **Ⅲ** |  |  |  |  |  |  |  |  |  |  |  |  |  |  |  |  |  |  |  |  |  |  |  |  |  |  |  |  |  |  |  |  |  |  |  |  |
| AHR | **-0.17564** | 0.000 | 0.72 | **-0.16233** | 0.000 | 0.57 | **-0.23107** | 0.000 | 0.70 | **-0.16105** | 0.000 | 0.71 | **-0.17547** | 0.000 | 0.79 | **-0.18833** | 0.000 | 0.80 | **-0.14846** | 0.000 | 0.83 | **-0.17971** | 0.000 | 0.78 | **-0.16500** | 0.000 | 0.75 | **-0.18457** | 0.000 | 0.81 | **-0.13419** | 0.000 | 0.80 | **-0.16944** | 0.000 | 0.78 |
| TR(＞25 miles) | **-0.00665** | 0.002 |  | **-0.02364** | 0.000 |  | **-0.17504** | 0.000 |  | -0.00208 | 0.392 |  | **-0.10728** | 0.000 |  | -0.00235 | 0.932 |  | **-0.07527** | 0.002 |  | **-0.12589** | 0.000 |  | **-0.15119** | 0.000 |  | -0.03959 | 0.136 |  | **-0.10755** | 0.000 |  | **-0.16958** | 0.000 |  |
| T | **-0.00021** | 0.000 |  | **-0.00011** | 0.000 |  | **-0.00021** | 0.000 |  | **-0.00023** | 0.000 |  | **-0.00018** | 0.000 |  | **-0.00020** | 0.000 |  | **-0.00017** | 0.000 |  | **-0.00018** | 0.000 |  | **-0.00019** | 0.000 |  | **-0.00021** | 0.000 |  | **-0.00020** | 0.000 |  | **-0.00019** | 0.000 |  |
| H | **-0.00004** | 0.000 |  | **0.00013** | 0.000 |  | **-0.00005** | 0.000 |  | **-0.00005** | 0.000 |  | **-0.00002** | 0.000 |  | **0.00004** | 0.010 |  | **0.00004** | 0.000 |  | **-0.00002** | 0.003 |  | **-0.00002** | 0.000 |  | 0.00003 | 0.065 |  | 0.00002 | 0.180 |  | **-0.00001** | 0.014 |  |
| WS | **0.00023** | 0.000 |  | **-0.00042** | 0.000 |  | **0.00034** | 0.000 |  | **0.00029** | 0.000 |  | -0.00001 | 0.710 |  | **-0.00016** | 0.017 |  | 0.00002 | 0.714 |  | 0.00001 | 0.813 |  | 0.00000 | 0.843 |  | **-0.00013** | 0.034 |  | 0.00004 | 0.395 |  | 0.00001 | 0.699 |  |
| AP | 0.00001 | 0.965 |  | -0.00018 | 0.877 |  | **0.00245** | 0.000 |  | -0.00015 | 0.238 |  | **0.00093** | 0.000 |  | **0.00347** | 0.000 |  | **0.00131** | 0.000 |  | **0.00081** | 0.000 |  | **0.00092** | 0.000 |  | **0.00356** | 0.000 |  | **0.00114** | 0.000 |  | **0.00080** | 0.000 |  |
| PPTN | -0.00024 | 0.441 |  | -0.00052 | 0.430 |  | **-0.01978** | 0.000 |  | 0.00004 | 0.903 |  | **0.00066** | 0.009 |  | -0.00101 | 0.052 |  | **-0.03022** | 0.000 |  | **0.00135** | 0.000 |  | **0.00067** | 0.008 |  | -0.00098 | 0.052 |  | **-0.02706** | 0.000 |  | **0.00126** | 0.000 |  |
| constant | 0.16227 | 0.000 |  | 0.15716 | 0.000 |  | 0.13791 | 0.000 |  | 0.15518 | 0.000 |  | 0.13384 | 0.000 |  | 0.07320 | 0.018 |  | 0.10083 | 0.000 |  | 0.13932 | 0.000 |  | 0.12781 | 0.000 |  | 0.06957 | 0.020 |  | 0.09794 | 0.000 |  | 0.13299 | 0.000 |  |
| **Ⅳ** |  |  |  |  |  |  |  |  |  |  |  |  |  |  |  |  |  |  |  |  |  |  |  |  |  |  |  |  |  |  |  |  |  |  |  |  |
| AHR | **-0.07838** | 0.000 | 0.57 | -0.01672 | 0.299 | 0.63 | **-0.06006** | 0.000 | 0.61 | **-0.08741** | 0.000 | 0.56 | **-0.06836** | 0.000 | 0.70 | -0.00083 | 0.949 | 0.74 | -0.00074 | 0.891 | 0.73 | **-0.07622** | 0.000 | 0.71 | **-0.08920** | 0.000 | 0.45 | **-0.02642** | 0.035 | 0.48 | **-0.04531** | 0.000 | 0.40 | **-0.09727** | 0.000 | 0.44 |
| TR(＞25 miles) | **-0.11241** | 0.007 |  | **-0.06271** | 0.000 |  | **-0.01516** | 0.000 |  | **-0.02487** | 0.000 |  | **-0.02193** | 0.000 |  | **-0.02393** | 0.000 |  | **-0.01490** | 0.000 |  | **-0.02042** | 0.000 |  | **-0.02473** | 0.000 |  | **-0.01807** | 0.000 |  | **-0.01781** | 0.000 |  | **-0.02553** | 0.000 |  |
| AVD | **-0.78474** | 0.000 |  | **-2.96161** | 0.000 |  | **-0.83313** | 0.000 |  | **-0.47063** | 0.000 |  | **-0.89330** | 0.000 |  | **-2.03829** | 0.000 |  | **-1.60389** | 0.000 |  | **-0.76971** | 0.000 |  | **-0.71302** | 0.000 |  | **-1.83827** | 0.000 |  | **-1.11926** | 0.000 |  | **-0.59441** | 0.000 |  |
| T | **0.00011** | 0.000 |  | **0.00014** | 0.000 |  | **0.00011** | 0.000 |  | **0.00014** | 0.000 |  | **0.00016** | 0.000 |  | **0.00012** | 0.000 |  | **0.00006** | 0.003 |  | **0.00016** | 0.000 |  | **0.00019** | 0.000 |  | **0.00016** | 0.000 |  | **0.00008** | 0.000 |  | **0.00020** | 0.000 |  |
| H | -0.00001 | 0.235 |  | 0.00002 | 0.442 |  | **0.00010** | 0.000 |  | **-0.00003** | 0.000 |  | 0.00000 | 0.718 |  | **-0.00005** | 0.003 |  | 0.00001 | 0.569 |  | 0.00001 | 0.126 |  | **-0.00002** | 0.000 |  | **-0.00008** | 0.000 |  | -0.00001 | 0.393 |  | **-0.00001** | 0.017 |  |
| WS | **-0.00040** | 0.000 |  | **-0.00048** | 0.000 |  | **-0.00011** | 0.015 |  | **-0.00045** | 0.000 |  | **-0.00018** | 0.000 |  | **-0.00048** | 0.000 |  | -0.00001 | 0.896 |  | **-0.00019** | 0.000 |  | **-0.00015** | 0.000 |  | **-0.00035** | 0.000 |  | -0.00002 | 0.729 |  | **-0.00016** | 0.000 |  |
| AP | -0.00086 | 0.050 |  | -0.00279 | 0.165 |  | **0.01034** | 0.000 |  | **-0.00225** | 0.000 |  | -0.00060 | 0.080 |  | **-0.00637** | 0.000 |  | **0.00588** | 0.000 |  | **-0.00112** | 0.003 |  | -0.00034 | 0.294 |  | **-0.00587** | 0.000 |  | **0.00489** | 0.000 |  | -0.00061 | 0.082 |  |
| PPTN | **-0.00135** | 0.002 |  | **-0.00436** | 0.007 |  | **-0.01300** | 0.000 |  | -0.00040 | 0.374 |  | **-0.00146** | 0.000 |  | **-0.00460** | 0.000 |  | -0.00359 | 0.374 |  | **-0.00131** | 0.000 |  | -0.00042 | 0.195 |  | **-0.00328** | 0.009 |  | -0.00197 | 0.609 |  | -0.00027 | 0.427 |  |
| constant | 0.10784 | 0.000 |  | 0.13187 | 0.030 |  | -0.19579 | 0.000 |  | 0.16126 | 0.000 |  | 0.09404 | 0.000 |  | 0.22603 | 0.000 |  | -0.11782 | 0.000 |  | 0.11397 | 0.000 |  | 0.10199 | 0.000 |  | 0.22825 | 0.000 |  | -0.06085 | 0.042 |  | 0.11515 | 0.000 |  |
| **Ⅴ** |  |  |  |  |  |  |  |  |  |  |  |  |  |  |  |  |  |  |  |  |  |  |  |  |  |  |  |  |  |  |  |  |  |  |  |  |
| AHR | -0.01094 | 0.120 | 0.19 | **-0.09494** | 0.000 | 0.34 | **-0.12050** | 0.000 | 0.32 | **-0.02197** | 0.009 | 0.20 | **-0.06394** | 0.000 | 0.19 | **-0.08384** | 0.000 | 0.49 | **-0.20615** | 0.000 | 0.31 | **-0.06643** | 0.000 | 0.21 | **-0.09148** | 0.000 | 0.20 | **-0.06774** | 0.000 | 0.41 | **-0.24523** | 0.000 | 0.19 | **-0.09696** | 0.000 | 0.20 |
| TR(＞25 miles) | **-0.06269** | 0.000 |  | **0.04254** | 0.000 |  | **-0.09791** | 0.000 |  | **-0.07074** | 0.000 |  | **-0.06416** | 0.000 |  | **0.03270** | 0.000 |  | **-0.08415** | 0.000 |  | **-0.06542** | 0.000 |  | **-0.09551** | 0.000 |  | -0.00592 | 0.507 |  | **-0.11138** | 0.000 |  | **-0.09771** | 0.000 |  |
| AVD | **-0.00001** | 0.000 |  | **-0.00003** | 0.000 |  | **-1.09700** | 0.000 |  | -0.03958 | 0.272 |  | **-0.56662** | 0.000 |  | **-0.00002** | 0.000 |  | **-0.87606** | 0.000 |  | **-0.65239** | 0.000 |  | **-0.73128** | 0.000 |  | **-0.00001** | 0.000 |  | **-1.04000** | 0.000 |  | **-0.81738** | 0.000 |  |
| T | **-0.00012** | 0.000 |  | **0.00036** | 0.000 |  | **-0.00036** | 0.000 |  | **-0.00028** | 0.000 |  | **-0.00023** | 0.000 |  | **-0.00013** | 0.000 |  | **-0.00022** | 0.000 |  | **-0.00026** | 0.000 |  | **-0.00024** | 0.000 |  | **-0.00013** | 0.000 |  | **-0.00021** | 0.000 |  | **-0.00027** | 0.000 |  |
| H | **0.00014** | 0.000 |  | **0.00016** | 0.000 |  | **-0.00013** | 0.000 |  | **0.00016** | 0.000 |  | **0.00007** | 0.000 |  | **0.00022** | 0.000 |  | -0.00003 | 0.091 |  | **0.00010** | 0.000 |  | **0.00007** | 0.000 |  | **0.00022** | 0.000 |  | -0.00002 | 0.294 |  | **0.00009** | 0.000 |  |
| WS | **-0.00092** | 0.000 |  | **-0.00208** | 0.000 |  | **-0.00082** | 0.000 |  | **-0.00066** | 0.000 |  | **-0.00070** | 0.000 |  | **-0.00141** | 0.000 |  | **-0.00071** | 0.000 |  | **-0.00059** | 0.000 |  | **-0.00074** | 0.000 |  | **-0.00149** | 0.000 |  | **-0.00070** | 0.000 |  | **-0.00064** | 0.000 |  |
| AP | **-0.00490** | 0.000 |  | **0.00527** | 0.008 |  | **-0.00177** | 0.001 |  | **-0.00732** | 0.000 |  | **-0.00321** | 0.000 |  | **-0.00990** | 0.000 |  | **-0.00060** | 0.201 |  | **-0.00503** | 0.000 |  | **-0.00328** | 0.000 |  | **-0.00849** | 0.000 |  | -0.00063 | 0.160 |  | **-0.00543** | 0.000 |  |
| PPTN | **-0.00199** | 0.000 |  | **0.00199** | 0.030 |  | **-0.00996** | 0.002 |  | **-0.00324** | 0.000 |  | **-0.00244** | 0.000 |  | **-0.00438** | 0.000 |  | **-0.01048** | 0.000 |  | **-0.00277** | 0.000 |  | **-0.00220** | 0.000 |  | **-0.00413** | 0.000 |  | **-0.01198** | 0.000 |  | **-0.00231** | 0.000 |  |
| constant | 0.19385 | 0.000 |  | -0.08917 | 0.137 |  | 0.15758 | 0.000 |  | 0.28188 | 0.000 |  | 0.15981 | 0.000 |  | 0.37689 | 0.000 |  | 0.12740 | 0.000 |  | 0.21556 | 0.000 |  | 0.17900 | 0.000 |  | 0.33301 | 0.000 |  | 0.14451 | 0.000 |  | 0.24609 | 0.000 |  |
| **Ⅵ** |  |  |  |  |  |  |  |  |  |  |  |  |  |  |  |  |  |  |  |  |  |  |  |  |  |  |  |  |  |  |  |  |  |  |  |  |
| AHR | **-0.23521** | 0.000 | 0.12 | 0.04642 | 0.813 | 0.19 | -0.09255 | 0.411 | 0.35 | **-0.15502** | 0.003 | 0.17 | -0.07117 | 0.080 | 0.20 | -0.09058 | 0.597 | 0.14 | **-0.58996** | 0.000 | 0.34 | **-0.10246** | 0.024 | 0.26 | **-0.17345** | 0.000 | 0.14 | -0.12589 | 0.469 | 0.08 | **-0.85469** | 0.000 | 0.31 | **-0.19175** | 0.000 | 0.18 |
| TR(＞25 miles) | **-0.16084** | 0.000 |  | **-0.37686** | 0.000 |  | -0.03229 | 0.511 |  | **-0.10620** | 0.000 |  | **-0.11506** | 0.000 |  | **-0.36953** | 0.000 |  | -0.04695 | 0.305 |  | **-0.05577** | 0.029 |  | 0.00293 | 0.897 |  | -0.21285 | 0.011 |  | 0.03307 | 0.473 |  | **0.05986** | 0.024 |  |
| AVD | **-0.54604** | 0.023 |  | 0.04806 | 0.976 |  | **-8.02352** | 0.000 |  | -0.21254 | 0.403 |  | **-1.57923** | 0.000 |  | 0.82762 | 0.553 |  | **-6.41215** | 0.000 |  | **-1.62903** | 0.000 |  | **-0.49435** | 0.020 |  | 1.31551 | 0.353 |  | **-5.57981** | 0.000 |  | -0.56759 | 0.013 |  |
| T | **-0.00053** | 0.000 |  | -0.00019 | 0.236 |  | **0.00095** | 0.000 |  | **-0.00061** | 0.000 |  | **-0.00068** | 0.000 |  | **-0.00049** | 0.000 |  | **0.00047** | 0.000 |  | **-0.00074** | 0.000 |  | **-0.00061** | 0.000 |  | **-0.00043** | 0.003 |  | **0.00038** | 0.004 |  | **-0.00067** | 0.000 |  |
| H | **0.00034** | 0.000 |  | **-0.00047** | 0.000 |  | **0.00057** | 0.000 |  | **0.00051** | 0.000 |  | **0.00035** | 0.000 |  | 0.00003 | 0.772 |  | **0.00053** | 0.000 |  | **0.00041** | 0.000 |  | **0.00047** | 0.000 |  | 0.00021 | 0.071 |  | **0.00045** | 0.000 |  | **0.00053** | 0.000 |  |
| WS | 0.00003 | 0.849 |  | 0.00120 | 0.131 |  | **-0.00274** | 0.000 |  | **0.00087** | 0.000 |  | 0.00026 | 0.074 |  | 0.00074 | 0.284 |  | **-0.00183** | 0.000 |  | **0.00080** | 0.000 |  | **0.00037** | 0.013 |  | 0.00061 | 0.384 |  | **-0.00145** | 0.000 |  | **0.00077** | 0.000 |  |
| AP | **0.00143** | 0.043 |  | **-0.01824** | 0.011 |  | **-0.01094** | 0.000 |  | **0.00576** | 0.000 |  | 0.00091 | 0.135 |  | **-0.01401** | 0.025 |  | **-0.00875** | 0.000 |  | **0.00505** | 0.000 |  | 0.00019 | 0.759 |  | **-0.01861** | 0.004 |  | **-0.00991** | 0.000 |  | **0.00472** | 0.000 |  |
| PPTN | **-0.01820** | 0.000 |  | -0.00276 | 0.750 |  | **-0.07157** | 0.001 |  | **-0.01932** | 0.000 |  | **-0.01064** | 0.000 |  | -0.01277 | 0.093 |  | **-0.03973** | 0.049 |  | **-0.01001** | 0.000 |  | **-0.01113** | 0.000 |  | **-0.02285** | 0.003 |  | -0.01186 | 0.558 |  | **-0.00964** | 0.000 |  |
| constant | 0.23409 | 0.000 |  | 0.68206 | 0.000 |  | 0.32903 | 0.000 |  | 0.00961 | 0.863 |  | 0.07137 | 0.001 |  | 0.59646 | 0.006 |  | 0.39157 | 0.000 |  | -0.06883 | 0.009 |  | 0.06395 | 0.003 |  | 0.68095 | 0.002 |  | 0.45559 | 0.000 |  | -0.08797 | 0.001 |  |

AHR, the proportion of daily residents at home; TR, Daily trips per capita; AVD, daily administered vaccination dose per capita; T, temperature; H, humidity; WS, wind speed; AP, air pressure; PPTN, precipitation
