# Supplementary material for: A panel regression analysis for the COVID-19 epidemic in the United States
Source: PLoS One. 2022 Aug 19;17(8):e0273344. doi: 10.1371/journal.pone.0273344 (PMC9390909; doi:10.1371/journal.pone.0273344)
Supplement: S3 Table — (DOCX) [file pone.0273344.s003.docx]

**S3 Table. Multivariate analysis of influencing factors - IR postponed for 3, 7, 10 days, TR as independent variable**

| **Variables** | **IR postponed for 3 days** | | | | | | | | | | | | **IR postponed for 7 days** | | | | | | | | | | | | **IR postponed for 10 days** | | | | | | | | | | | |
| --- | --- | --- | --- | --- | --- | --- | --- | --- | --- | --- | --- | --- | --- | --- | --- | --- | --- | --- | --- | --- | --- | --- | --- | --- | --- | --- | --- | --- | --- | --- | --- | --- | --- | --- | --- | --- |
|  | **50 states** | | | **the first category** | | | **the second category** | | | **the third category** | | | **50 states** | | | **the first category** | | | **the second category** | | | **the third category** | | | **50 states** | | | **the first category** | | | **the second category** | | | **the third category** | | |
|  | **Coef.** | ***P*** | **R^2^** | **Coef.** | ***P*** | **R^2^** | **Coef.** | ***P*** | **R^2^** | **Coef.** | ***P*** | **R^2^** | **Coef.** | ***P*** | **R^2^** | **Coef.** | ***P*** | **R^2^** | **Coef.** | ***P*** | **R^2^** | **Coef.** | ***P*** | **R^2^** | **Coef.** | ***P*** | **R^2^** | **Coef.** | ***P*** | **R^2^** | **Coef.** | ***P*** | **R^2^** | **Coef.** | ***P*** | **R^2^** |
| **Unsegmented** |  |  |  |  |  |  |  |  |  |  |  |  |  |  |  |  |  |  |  |  |  |  |  |  |  |  |  |  |  |  |  |  |  |  |  |  |
| AHR | **-8.50E-03** | 0.000 | 0.27 | **-3.20E-01** | 0.000 | 0.28 | **-3.70E-01** | 0.000 | 0.48 | **-8.20E-02** | 0.000 | 0.36 | **-1.70E-01** | 0.000 | 0.43 | **-1.30E-02** | 0.000 | 0.28 | **-4.40E-01** | 0.000 | 0.58 | **-1.20E-01** | 0.000 | 0.45 | **-1.60E-01** | 0.000 | 0.41 | **-2.00E-01** | 0.000 | 0.33 | **-4.60E-01** | 0.000 | 0.58 | **-1.00E-01** | 0.000 | 0.43 |
| TR | **2.40E-04** | 0.000 |  | **-1.90E-02** | 0.000 |  | **-1.10E-02** | 0.000 |  | **5.20E-03** | 0.000 |  | **1.70E-03** | 0.000 |  | -7.50E-05 | 0.481 |  | **-1.40E-02** | 0.000 |  | **4.80E-03** | 0.000 |  | **2.50E-03** | 0.000 |  | **-4.20E-03** | 0.004 |  | **-1.40E-02** | 0.000 |  | **5.80E-03** | 0.000 |  |
| AVD | **-1.40E-01** | 0.000 |  | **-2.80E+00** | 0.000 |  | **-2.40E+00** | 0.000 |  | **-1.70E+00** | 0.000 |  | **-2.40E+00** | 0.000 |  | **-2.20E-01** | 0.000 |  | **-2.60E+00** | 0.000 |  | **-2.30E+00** | 0.000 |  | **-2.40E+00** | 0.000 |  | **-2.90E+00** | 0.000 |  | **-2.70E+00** | 0.000 |  | **-2.20E+00** | 0.000 |  |
| T | **-2.90E-05** | 0.000 |  | **-3.60E-04** | 0.000 |  | **-4.50E-04** | 0.000 |  | **-4.90E-04** | 0.000 |  | **-5.00E-04** | 0.000 |  | **-3.20E-05** | 0.000 |  | **-5.10E-04** | 0.000 |  | **-5.00E-04** | 0.000 |  | **-4.80E-04** | 0.000 |  | **-5.20E-04** | 0.000 |  | **-5.00E-04** | 0.000 |  | **-4.80E-04** | 0.000 |  |
| H | -1.00E-06 | 0.113 |  | **-8.40E-05** | 0.004 |  | **-5.10E-05** | 0.012 |  | **7.40E-05** | 0.000 |  | 1.30E-05 | 0.098 |  | -1.30E-06 | 0.496 |  | **-4.00E-05** | 0.031 |  | **4.70E-05** | 0.000 |  | **3.30E-05** | 0.000 |  | **8.20E-05** | 0.001 |  | -3.60E-05 | 0.054 |  | **6.80E-05** | 0.000 |  |
| WS | -1.10E-06 | 0.738 |  | **-9.20E-04** | 0.000 |  | **-8.70E-04** | 0.000 |  | 3.20E-05 | 0.533 |  | **-1.20E-04** | 0.002 |  | **-2.20E-05** | 0.039 |  | **-3.10E-04** | 0.000 |  | 6.10E-05 | 0.185 |  | **-1.00E-04** | 0.008 |  | **-8.40E-04** | 0.000 |  | **-1.80E-04** | 0.022 |  | 4.50E-05 | 0.333 |  |
| AP | 2.30E-05 | 0.064 |  | **-2.30E-02** | 0.000 |  | **-3.30E-03** | 0.000 |  | **-1.00E-03** | 0.026 |  | **-2.50E-03** | 0.000 |  | **-1.50E-03** | 0.000 |  | **-2.10E-03** | 0.000 |  | **-2.30E-03** | 0.000 |  | **-3.00E-03** | 0.000 |  | **-2.70E-02** | 0.000 |  | **-3.00E-03** | 0.000 |  | **-2.70E-03** | 0.000 |  |
| PPTN | **-1.10E-04** | 0.012 |  | 2.70E-03 | 0.112 |  | **-2.40E-02** | 0.000 |  | **-4.20E-03** | 0.000 |  | **-2.20E-03** | 0.000 |  | -7.10E-05 | 0.517 |  | **-2.00E-02** | 0.000 |  | **-2.60E-03** | 0.000 |  | **-2.30E-03** | 0.000 |  | **-4.00E-03** | 0.008 |  | **-1.80E-02** | 0.000 |  | **-2.60E-03** | 0.000 |  |
| constant | 3.10E-03 | 0.000 |  | 8.90E-01 | 0.000 |  | 2.70E-01 | 0.000 |  | 7.70E-02 | 0.000 |  | 1.60E-01 | 0.000 |  | 5.10E-02 | 0.000 |  | 2.70E-01 | 0.000 |  | 1.30E-01 | 0.000 |  | 1.70E-01 | 0.000 |  | 9.20E-01 | 0.000 |  | 3.00E-01 | 0.000 |  | 1.30E-01 | 0.000 |  |
| **Segmented** |  |  |  |  |  |  |  |  |  |  |  |  |  |  |  |  |  |  |  |  |  |  |  |  |  |  |  |  |  |  |  |  |  |  |  |  |
| **Ⅰ** |  |  |  |  |  |  |  |  |  |  |  |  |  |  |  |  |  |  |  |  |  |  |  |  |  |  |  |  |  |  |  |  |  |  |  |  |
| AHR | **-7.60E-03** | 0.004 | 0.80 | **-1.30E-03** | 0.001 | 0.49 | **-1.00E-04** | 0.018 | 0.82 | **-7.50E-04** | 0.000 | 0.48 | **-6.50E-04** | 0.000 | 0.39 | **-3.70E-04** | 0.001 | 0.88 | 8.40E-05 | 0.222 | 0.76 | -6.00E-05 | 0.181 | 0.68 | 7.30E-05 | 0.059 | 0.73 | **-2.70E-04** | 0.011 | 0.90 | **6.60E-05** | -0.001 | 0.76 | -5.70E-05 | 0.190 | 0.74 |
| TR | **-6.10E-03** | 0.000 |  | **-1.90E-04** | 0.000 |  | **-4.80E-05** | 0.000 |  | **-1.30E-04** | 0.000 |  | **-1.20E-04** | 0.000 |  | **-8.90E-05** | 0.000 |  | **-8.70E-05** | 0.000 |  | **-5.70E-05** | 0.000 |  | **-5.50E-05** | 0.000 |  | **-9.50E-05** | 0.000 |  | **-1.30E-05** | 0.015 |  | **-5.80E-05** | 0.000 |  |
| T | **-4.10E-05** | 0.000 |  | **-2.90E-06** | 0.019 |  | -1.40E-07 | 0.506 |  | **-2.30E-06** | 0.000 |  | -2.70E-07 | 0.086 |  | -6.90E-08 | 0.851 |  | 3.40E-07 | 0.546 |  | -6.70E-08 | 0.693 |  | -2.70E-07 | 0.071 |  | 5.20E-07 | 0.157 |  | -2.10E-07 | 0.732 |  | 3.10E-07 | 0.062 |  |
| H | 5.60E-06 | 0.289 |  | 6.40E-07 | 0.214 |  | 4.10E-08 | 0.647 |  | **5.20E-07** | 0.004 |  | 1.30E-07 | 0.085 |  | **-5.70E-07** | 0.000 |  | -3.90E-07 | 0.087 |  | **-2.40E-07** | 0.004 |  | **-1.60E-07** | 0.021 |  | **-3.90E-07** | 0.012 |  | **-5.60E-07** | 0.018 |  | 1.50E-07 | 0.062 |  |
| WS | **9.50E-05** | 0.001 |  | 1.60E-06 | 0.615 |  | -2.20E-07 | 0.685 |  | **2.80E-06** | 0.001 |  | 4.10E-07 | 0.182 |  | **-3.50E-06** | 0.000 |  | **5.60E-06** | 0.000 |  | **8.90E-07** | 0.027 |  | 7.20E-07 | 0.051 |  | **-2.90E-06** | 0.003 |  | **4.60E-06** | 0.002 |  | **1.00E-06** | 0.007 |  |
| AP | **3.60E-04** | 0.009 |  | 2.30E-05 | 0.728 |  | -3.70E-06 | 0.664 |  | 1.40E-05 | 0.124 |  | 2.90E-06 | 0.465 |  | -2.70E-05 | 0.174 |  | **1.00E-04** | 0.001 |  | **-8.60E-06** | 0.049 |  | -4.60E-06 | 0.272 |  | 2.20E-05 | 0.276 |  | 6.00E-05 | 0.057 |  | 4.00E-06 | 0.331 |  |
| PPTN | **-2.10E-03** | 0.000 |  | **-1.80E-04** | 0.000 |  | -3.60E-05 | 0.074 |  | **-4.80E-05** | 0.000 |  | -3.80E-06 | 0.370 |  | -1.30E-06 | 0.929 |  | 2.10E-05 | 0.693 |  | **-3.20E-05** | 0.000 |  | **-3.70E-05** | 0.000 |  | 2.20E-06 | 0.878 |  | -8.40E-05 | 0.122 |  | **-3.50E-05** | 0.000 |  |
| constant | 1.60E-02 | 0.000 |  | 3.30E-04 | 0.861 |  | 3.00E-04 | 0.179 |  | 2.70E-04 | 0.284 |  | 4.70E-04 | 0.000 |  | 1.30E-03 | 0.026 |  | -2.40E-03 | 0.003 |  | 4.90E-04 | 0.000 |  | 3.50E-04 | 0.003 |  | -2.40E-04 | 0.676 |  | -8.10E-04 | 0.301 |  | 8.50E-05 | 0.467 |  |
| **Ⅱ** |  |  |  |  |  |  |  |  |  |  |  |  |  |  |  |  |  |  |  |  |  |  |  |  |  |  |  |  |  |  |  |  |  |  |  |  |
| AHR | **-7.70E-02** | 0.000 | 0.14 | **-1.10E-01** | 0.000 | 0.70 | **-9.30E-04** | 0.000 | 0.37 | **-1.20E-03** | 0.000 | 0.10 | **-1.40E-02** | 0.000 | 0.21 | **-7.40E-04** | 0.002 | 0.36 | -1.60E-04 | 0.287 | 0.21 | **-3.50E-04** | 0.000 | 0.23 | **-1.70E-02** | 0.000 | 0.25 | **-6.50E-04** | 0.008 | 0.38 | **-3.00E-04** | 0.048 | 0.25 | **-3.60E-04** | 0.000 | 0.27 |
| TR | **-9.00E-03** | 0.000 |  | **-1.80E-02** | 0.000 |  | **-1.50E-04** | 0.000 |  | **-1.40E-04** | 0.000 |  | **-4.10E-03** | 0.000 |  | **-1.80E-04** | 0.000 |  | **-7.60E-05** | 0.000 |  | **-8.60E-05** | 0.000 |  | **-4.60E-03** | 0.000 |  | **-1.80E-04** | 0.000 |  | **-9.10E-05** | 0.000 |  | **-9.40E-05** | 0.000 |  |
| T | **2.50E-05** | 0.000 |  | **4.10E-04** | 0.000 |  | **2.20E-06** | 0.000 |  | **-3.20E-07** | 0.038 |  | **6.80E-05** | 0.000 |  | **2.30E-06** | 0.000 |  | **1.30E-06** | 0.000 |  | **1.20E-06** | 0.000 |  | **8.00E-05** | 0.000 |  | **3.00E-06** | 0.000 |  | **1.50E-06** | 0.000 |  | **1.60E-06** | 0.000 |  |
| H | **-2.50E-05** | 0.000 |  | -3.00E-05 | 0.138 |  | 3.00E-07 | 0.322 |  | -2.50E-07 | 0.099 |  | **9.10E-06** | 0.010 |  | **6.20E-07** | 0.039 |  | -2.90E-07 | 0.299 |  | **2.30E-07** | 0.003 |  | 2.50E-06 | 0.488 |  | 4.60E-07 | 0.152 |  | -5.20E-07 | 0.064 |  | 1.40E-07 | 0.075 |  |
| WS | **2.20E-04** | 0.000 |  | 1.40E-04 | 0.124 |  | -7.30E-07 | 0.549 |  | **4.60E-06** | 0.000 |  | **-9.10E-05** | 0.000 |  | -7.10E-07 | 0.606 |  | -1.10E-06 | 0.328 |  | **-1.80E-06** | 0.000 |  | **-1.10E-04** | 0.000 |  | -7.90E-07 | 0.585 |  | -1.20E-06 | 0.284 |  | **-2.30E-06** | 0.000 |  |
| AP | -3.20E-05 | 0.792 |  | **8.60E-03** | 0.000 |  | **-7.30E-06** | 0.001 |  | **-3.00E-05** | 0.004 |  | -1.30E-04 | 0.076 |  | **7.90E-05** | 0.011 |  | -3.00E-06 | 0.156 |  | 4.90E-06 | 0.378 |  | -1.00E-04 | 0.172 |  | **1.10E-04** | 0.000 |  | -3.00E-06 | 0.164 |  | **1.20E-05** | 0.040 |  |
| PPTN | **1.90E-03** | 0.000 |  | **1.60E-03** | 0.015 |  | 2.00E-06 | 0.963 |  | **3.90E-05** | 0.000 |  | -1.10E-04 | 0.611 |  | 5.10E-06 | 0.608 |  | -6.20E-06 | 0.878 |  | -8.30E-06 | 0.090 |  | 4.40E-05 | 0.844 |  | 1.10E-05 | 0.300 |  | 3.00E-05 | 0.467 |  | -4.90E-06 | 0.330 |  |
| constant | 5.40E-02 | 0.000 |  | -2.00E-01 | 0.002 |  | 8.20E-04 | 0.000 |  | 1.70E-03 | 0.000 |  | 2.50E-02 | 0.000 |  | -1.70E-03 | 0.063 |  | 3.80E-04 | 0.000 |  | 2.20E-04 | 0.186 |  | 2.60E-02 | 0.000 |  | -2.90E-03 | 0.004 |  | 4.70E-04 | 0.000 |  | 3.70E-05 | 0.829 |  |
| **Ⅲ** |  |  |  |  |  |  |  |  |  |  |  |  |  |  |  |  |  |  |  |  |  |  |  |  |  |  |  |  |  |  |  |  |  |  |  |  |
| AHR | -6.30E-04 | 0.713 | 0.65 | -1.50E-03 | 0.714 | 0.48 | **-8.40E-04** | 0.026 | 0.71 | -4.60E-05 | 0.661 | 0.56 | 7.40E-06 | 0.901 | 0.67 | **-1.30E-04** | 0.000 | 0.71 | -1.70E-04 | 0.534 | 0.73 | 8.50E-05 | 0.494 | 0.48 | **-1.80E-01** | 0.000 | 0.81 | **-7.30E-03** | 0.000 | 0.80 | 6.00E-05 | 0.064 | 0.74 | **-8.10E-03** | 0.000 | 0.78 |
| TR | **1.70E-03** | 0.000 |  | **-3.60E-03** | 0.007 |  | 1.50E-05 | 0.774 |  | **1.90E-04** | 0.000 |  | **2.50E-04** | 0.000 |  | 6.60E-05 | 0.140 |  | **1.40E-04** | 0.000 |  | **2.20E-04** | 0.000 |  | **7.80E-03** | 0.000 |  | **1.40E-04** | 0.000 |  | **1.50E-04** | 0.000 |  | **3.80E-04** | 0.000 |  |
| T | **-3.80E-04** | 0.000 |  | **-2.20E-04** | 0.000 |  | **-1.80E-05** | 0.000 |  | **-1.50E-05** | 0.000 |  | **-1.40E-05** | 0.000 |  | **-1.20E-05** | 0.000 |  | **-1.20E-05** | 0.000 |  | **-1.50E-05** | 0.000 |  | **-2.00E-04** | 0.000 |  | **-8.00E-06** | 0.000 |  | **-1.30E-05** | 0.000 |  | **-6.60E-06** | 0.000 |  |
| H | **-4.90E-05** | 0.000 |  | **1.30E-04** | 0.000 |  | **-1.90E-06** | 0.012 |  | **-1.30E-06** | 0.001 |  | **-6.00E-07** | 0.005 |  | **1.60E-06** | 0.027 |  | **1.40E-06** | 0.010 |  | **-1.30E-06** | 0.003 |  | 2.40E-06 | 0.563 |  | **1.20E-06** | 0.036 |  | 3.50E-07 | 0.528 |  | **1.00E-06** | 0.000 |  |
| WS | **3.90E-04** | 0.000 |  | **-3.50E-04** | 0.000 |  | **2.60E-05** | 0.000 |  | **1.90E-05** | 0.000 |  | **8.60E-06** | 0.000 |  | -2.60E-07 | 0.933 |  | **8.10E-06** | 0.000 |  | **2.00E-05** | 0.000 |  | **1.10E-04** | 0.000 |  | -4.00E-06 | 0.122 |  | **8.30E-06** | 0.000 |  | **5.50E-06** | 0.000 |  |
| AP | **3.60E-04** | 0.006 |  | **4.20E-03** | 0.001 |  | **1.40E-04** | 0.000 |  | **-4.60E-05** | 0.000 |  | **3.80E-05** | 0.000 |  | **2.60E-04** | 0.000 |  | **4.10E-05** | 0.001 |  | **-4.50E-05** | 0.000 |  | **8.30E-04** | 0.000 |  | **1.10E-04** | 0.003 |  | **3.20E-05** | 0.008 |  | **1.60E-05** | 0.000 |  |
| PPTN | -3.20E-04 | 0.365 |  | -7.20E-04 | 0.325 |  | **-1.30E-03** | 0.000 |  | **-5.00E-05** | 0.011 |  | **3.10E-05** | 0.013 |  | -2.60E-05 | 0.281 |  | **-1.40E-03** | 0.000 |  | **-4.70E-05** | 0.042 |  | **7.20E-04** | 0.003 |  | -2.60E-05 | 0.194 |  | **-1.30E-03** | 0.000 |  | **4.30E-05** | 0.000 |  |
| constant | 2.50E-02 | 0.000 |  | -8.50E-02 | 0.027 |  | -1.60E-03 | 0.005 |  | 2.00E-03 | 0.000 |  | -6.40E-04 | 0.000 |  | -6.60E-03 | 0.000 |  | -3.70E-04 | 0.371 |  | 1.80E-03 | 0.000 |  | 1.20E-01 | 0.000 |  | 3.00E-03 | 0.010 |  | -2.30E-04 | 0.492 |  | 5.00E-03 | 0.000 |  |
| **Ⅳ** |  |  |  |  |  |  |  |  |  |  |  |  |  |  |  |  |  |  |  |  |  |  |  |  |  |  |  |  |  |  |  |  |  |  |  |  |
| AHR | **-1.90E-02** | 0.000 | 0.62 | **-3.20E-03** | 0.000 | 0.56 | **-1.10E-03** | 0.044 | 0.57 | **-1.10E-01** | 0.000 | 0.89 | **-3.70E-02** | 0.000 | 0.74 | **-1.00E-01** | 0.000 | 0.77 | **-4.20E-03** | 0.000 | 0.76 | **-2.60E-02** | 0.000 | 0.75 | -5.30E-04 | 0.654 | 0.74 | -3.60E-04 | 0.547 | 0.75 | **-2.10E-03** | 0.000 | 0.76 | **-1.30E-02** | 0.006 | 0.75 |
| TR | **-8.20E-03** | 0.000 |  | **-7.70E-04** | 0.000 |  | **-3.70E-04** | 0.000 |  | **-1.20E-02** | 0.000 |  | **-8.20E-03** | 0.000 |  | **-1.00E-02** | 0.000 |  | **-3.70E-04** | 0.000 |  | **-8.10E-03** | 0.000 |  | **-6.60E-03** | 0.000 |  | **-1.90E-04** | 0.000 |  | **-2.90E-04** | 0.000 |  | **-2.10E-04** | 0.000 |  |
| AVD | **-4.30E-01** | 0.000 |  | **-5.50E-04** | 0.000 |  | **-3.10E-04** | 0.000 |  | 3.00E-07 | 0.716 |  | **-6.90E-01** | 0.000 |  | **-1.40E+00** | 0.000 |  | **-3.40E-02** | 0.000 |  | **-6.00E-01** | 0.000 |  | **-6.20E-01** | 0.000 |  | **-6.30E-02** | 0.000 |  | **-3.10E-02** | 0.000 |  | **-5.20E-01** | 0.000 |  |
| T | **1.20E-04** | 0.000 |  | **1.00E-05** | 0.000 |  | **3.30E-06** | 0.018 |  | **2.50E-04** | 0.000 |  | **1.40E-04** | 0.000 |  | **8.00E-05** | 0.000 |  | **2.40E-06** | 0.001 |  | **1.60E-04** | 0.000 |  | **1.70E-04** | 0.000 |  | **6.30E-06** | 0.000 |  | **2.70E-06** | 0.000 |  | **1.80E-04** | 0.000 |  |
| H | -1.20E-05 | 0.066 |  | -3.00E-06 | 0.067 |  | **3.10E-06** | 0.003 |  | **-3.70E-04** | 0.000 |  | 8.00E-06 | 0.111 |  | **-5.30E-05** | 0.001 |  | 4.40E-09 | 0.994 |  | **1.60E-05** | 0.007 |  | **-1.10E-05** | 0.027 |  | **-3.20E-06** | 0.000 |  | -8.80E-07 | 0.128 |  | -4.80E-06 | 0.401 |  |
| WS | **-4.50E-04** | 0.000 |  | -1.40E-05 | 0.160 |  | **-1.40E-05** | 0.000 |  | **-8.80E-04** | 0.000 |  | **-2.40E-04** | 0.000 |  | **-5.30E-04** | 0.000 |  | **-6.40E-06** | 0.003 |  | **-2.30E-04** | 0.000 |  | **-2.00E-04** | 0.000 |  | **-1.60E-05** | 0.000 |  | **-5.20E-06** | 0.013 |  | **-1.90E-04** | 0.000 |  |
| AP | -4.50E-04 | 0.283 |  | **4.30E-05** | 0.009 |  | 3.10E-06 | 0.738 |  | **2.20E-04** | 0.049 |  | -4.80E-04 | 0.131 |  | **-7.00E-03** | 0.000 |  | **2.10E-04** | 0.000 |  | -3.20E-04 | 0.351 |  | -3.60E-04 | 0.241 |  | **-2.20E-04** | 0.001 |  | **1.60E-04** | 0.000 |  | -2.20E-04 | 0.512 |  |
| PPTN | **-1.00E-03** | 0.015 |  | **-3.80E-04** | 0.004 |  | **-1.20E-03** | 0.000 |  | **1.40E-02** | 0.000 |  | **-1.50E-03** | 0.000 |  | **-5.20E-03** | 0.000 |  | 1.40E-04 | 0.346 |  | **-1.40E-03** | 0.000 |  | -5.40E-04 | 0.087 |  | **-1.20E-04** | 0.030 |  | 1.70E-04 | 0.265 |  | -4.80E-04 | 0.140 |  |
| constant | 6.70E-02 | 0.000 |  | 2.40E-03 | 0.000 |  | 1.80E-03 | 0.000 |  | 8.30E-02 | 0.000 |  | 7.00E-02 | 0.000 |  | 2.90E-01 | 0.000 |  | -2.40E-03 | 0.044 |  | 6.10E-02 | 0.000 |  | 5.20E-02 | 0.000 |  | 8.10E-03 | 0.000 |  | -1.90E-03 | 0.100 |  | 5.50E-02 | 0.000 |  |
| **Ⅴ** |  |  |  |  |  |  |  |  |  |  |  |  |  |  |  |  |  |  |  |  |  |  |  |  |  |  |  |  |  |  |  |  |  |  |  |  |
| AHR | **-0.04463** | 0.000 | 0.16 | **-0.00899** | 0.000 | 0.40 | **-0.35186** | 0.000 | 0.43 | 0.00075 | 0.083 | 0.19 | **-3.20E-03** | 0.000 | 0.18 | **-9.30E-03** | 0.000 | 0.34 | **-1.20E-02** | 0.000 | 0.34 | **-8.20E-02** | 0.000 | 0.19 | **-1.40E-01** | 0.000 | 0.20 | **-2.70E-03** | 0.000 | 0.45 | **-1.30E-02** | 0.000 | 0.39 | **-1.00E-01** | 0.000 | 0.21 |
| TR | **-0.00612** | 0.000 |  | **-0.00081** | 0.000 |  | **-0.01912** | 0.000 |  | **-0.00009** | 0.000 |  | **-1.60E-04** | 0.000 |  | **-8.00E-04** | 0.000 |  | **-5.20E-04** | 0.000 |  | **-2.40E-03** | 0.000 |  | **-5.40E-03** | 0.000 |  | **-4.40E-04** | 0.000 |  | **-5.10E-04** | 0.000 |  | **-2.50E-03** | 0.000 |  |
| AVD | -0.02328 | 0.501 |  | 0.00000 | 0.259 |  | **-1.04229** | 0.000 |  | **-0.00664** | 0.000 |  | **-2.50E-02** | 0.000 |  | -8.70E-07 | 0.427 |  | **-1.30E-02** | 0.000 |  | **-7.00E-01** | 0.000 |  | **-8.20E-01** | 0.000 |  | **-3.50E-07** | 0.000 |  | **-3.80E-02** | 0.000 |  | **-8.80E-01** | 0.000 |  |
| T | **-0.00025** | 0.000 |  | **0.00001** | 0.000 |  | **-0.00046** | 0.000 |  | **-0.00001** | 0.000 |  | **-8.90E-06** | 0.000 |  | **9.40E-06** | 0.000 |  | **-8.20E-06** | 0.000 |  | **-2.80E-04** | 0.000 |  | **-2.70E-04** | 0.000 |  | **-5.60E-06** | 0.000 |  | **-1.00E-05** | 0.000 |  | **-2.90E-04** | 0.000 |  |
| H | **0.00010** | 0.000 |  | **0.00000** | 0.000 |  | **-0.00016** | 0.000 |  | **0.00001** | 0.000 |  | **2.20E-06** | 0.000 |  | **3.80E-07** | 0.000 |  | -3.80E-07 | 0.502 |  | **1.00E-04** | 0.000 |  | **6.80E-05** | 0.000 |  | **6.00E-06** | 0.000 |  | **-1.20E-06** | 0.035 |  | **9.50E-05** | 0.000 |  |
| WS | **-0.00080** | 0.000 |  | **-0.00007** | 0.000 |  | **-0.00078** | 0.000 |  | **-0.00002** | 0.000 |  | **-2.10E-05** | 0.000 |  | **-7.30E-05** | 0.000 |  | **-2.50E-05** | 0.000 |  | **-5.60E-04** | 0.000 |  | **-7.00E-04** | 0.000 |  | **-4.00E-05** | 0.000 |  | **-2.30E-05** | 0.000 |  | **-6.10E-04** | 0.000 |  |
| AP | **-0.00532** | 0.000 |  | -0.00008 | 0.391 |  | **-0.00130** | 0.007 |  | **-0.00031** | 0.000 |  | **-1.00E-04** | 0.000 |  | -3.20E-05 | 0.760 |  | -5.70E-06 | 0.717 |  | **-4.90E-03** | 0.000 |  | **-3.20E-03** | 0.000 |  | **-2.40E-04** | 0.000 |  | -4.60E-06 | 0.764 |  | **-5.40E-03** | 0.000 |  |
| PPTN | **-0.00223** | 0.000 |  | **0.00016** | 0.000 |  | -0.00521 | 0.080 |  | **-0.00013** | 0.000 |  | **-8.00E-05** | 0.000 |  | **1.50E-04** | 0.002 |  | **-2.60E-04** | 0.006 |  | **-3.10E-03** | 0.000 |  | **-2.40E-03** | 0.000 |  | **-1.30E-04** | 0.000 |  | **-2.80E-04** | 0.003 |  | **-2.70E-03** | 0.000 |  |
| constant | 0.21942 | 0.000 |  | 0.00715 | 0.009 |  | 0.25442 | 0.000 |  | 0.01005 | 0.000 |  | 5.30E-03 | 0.000 |  | 5.80E-03 | 0.067 |  | 6.00E-03 | 0.000 |  | 2.10E-01 | 0.000 |  | 1.80E-01 | 0.000 |  | 1.00E-02 | 0.000 |  | 6.50E-03 | 0.000 |  | 2.30E-01 | 0.000 |  |
| **Ⅵ** |  |  |  |  |  |  |  |  |  |  |  |  |  |  |  |  |  |  |  |  |  |  |  |  |  |  |  |  |  |  |  |  |  |  |  |  |
| AHR | **-0.31078** | 0.000 | 0.13 | -0.01140 | 0.460 | 0.26 | **-0.01062** | 0.343 | 0.35 | **-0.01724** | 0.001 | 0.14 | -3.80E-02 | 0.350 | 0.20 | -1.60E-02 | 0.294 | 0.21 | **-5.90E-02** | 0.000 | 0.30 | -7.30E-02 | 0.108 | 0.26 | **-1.60E-01** | 0.000 | 0.14 | -2.60E-02 | 0.082 | 0.18 | **-8.60E-02** | 0.000 | 0.27 | **-1.80E-01** | 0.000 | 0.19 |
| TR | **0.00963** | 0.000 |  | **-0.00592** | 0.000 |  | **-0.00126** | 0.019 |  | **0.00076** | 0.002 |  | -2.90E-04 | 0.877 |  | **-5.80E-03** | 0.000 |  | **-2.20E-03** | 0.000 |  | **6.00E-03** | 0.004 |  | **5.40E-03** | 0.005 |  | **-5.30E-03** | 0.000 |  | **-2.10E-03** | 0.000 |  | **1.20E-02** | 0.000 |  |
| AVD | **-1.40555** | 0.000 |  | 0.14000 | 0.251 |  | **-0.53489** | 0.000 |  | **-0.10440** | 0.001 |  | **-1.70E+00** | 0.000 |  | 1.80E-01 | 0.123 |  | **-3.80E-01** | 0.000 |  | **-2.10E+00** | 0.000 |  | **-8.60E-01** | 0.001 |  | 2.20E-01 | 0.061 |  | **-3.30E-01** | 0.000 |  | **-1.30E+00** | 0.000 |  |
| T | **-0.00051** | 0.000 |  | 0.00001 | 0.367 |  | **0.00010** | 0.000 |  | **-0.00005** | 0.000 |  | **-6.90E-04** | 0.000 |  | -2.10E-05 | 0.089 |  | **4.80E-05** | 0.000 |  | **-7.10E-04** | 0.000 |  | **-5.90E-04** | 0.000 |  | -1.50E-05 | 0.230 |  | **3.40E-05** | 0.009 |  | **-6.10E-04** | 0.000 |  |
| H | **0.00030** | 0.000 |  | **-0.00004** | 0.000 |  | **0.00006** | 0.000 |  | **0.00005** | 0.000 |  | **3.40E-04** | 0.000 |  | 4.00E-06 | 0.692 |  | **5.50E-05** | 0.000 |  | **3.90E-04** | 0.000 |  | **4.60E-04** | 0.000 |  | 2.00E-05 | 0.055 |  | **4.50E-05** | 0.000 |  | **5.10E-04** | 0.000 |  |
| WS | 0.00007 | 0.683 |  | **0.00013** | 0.042 |  | **-0.00025** | 0.000 |  | **0.00012** | 0.000 |  | 2.40E-04 | 0.100 |  | 7.40E-05 | 0.221 |  | **-1.70E-04** | 0.000 |  | **9.20E-04** | 0.000 |  | **4.00E-04** | 0.006 |  | 8.00E-05 | 0.193 |  | **-1.10E-04** | 0.000 |  | **9.40E-04** | 0.000 |  |
| AP | **0.00178** | 0.012 |  | **-0.00167** | 0.003 |  | **-0.00084** | 0.000 |  | **0.00044** | 0.000 |  | 1.10E-03 | 0.064 |  | **-1.30E-03** | 0.016 |  | **-7.70E-04** | 0.000 |  | **5.40E-03** | 0.000 |  | 2.10E-04 | 0.735 |  | **-2.00E-03** | 0.000 |  | **-8.50E-04** | 0.000 |  | **4.50E-03** | 0.000 |  |
| PPTN | **-0.01650** | 0.000 |  | 0.00042 | 0.531 |  | **-0.00750** | 0.000 |  | **-0.00170** | 0.000 |  | **-1.00E-02** | 0.000 |  | -9.40E-04 | 0.154 |  | **-4.90E-03** | 0.014 |  | **-9.20E-03** | 0.000 |  | **-1.00E-02** | 0.000 |  | **-2.00E-03** | 0.003 |  | -1.80E-03 | 0.374 |  | **-8.10E-03** | 0.001 |  |
| constant | **0.20077** | 0.000 |  | 0.07635 | 0.000 |  | 0.02764 | 0.000 |  | -0.00275 | 0.620 |  | 2.70E-02 | 0.187 |  | 6.50E-02 | 0.001 |  | 4.10E-02 | 0.000 |  | -1.20E-01 | 0.000 |  | 3.90E-02 | 0.065 |  | 8.40E-02 | 0.000 |  | 4.90E-02 | 0.000 |  | -1.20E-01 | 0.000 |  |

AHR, the proportion of daily residents at home; TR, Daily trips per capita; AVD, daily administered vaccination dose per capita; T, temperature; H, humidity; WS, wind speed; AP, air pressure; PPTN, precipitation
